# Duchenne muscular dystrophy: disease mechanism and therapeutic strategies

**DOI:** 10.3389/fphys.2023.1183101

**Published:** 2023-06-26

**Authors:** Addeli Bez Batti Angulski, Nora Hosny, Houda Cohen, Ashley A. Martin, Dongwoo Hahn, Jack Bauer, Joseph M. Metzger

**Affiliations:** Department of Integrative Biology and Physiology, University of Minnesota Medical School, Minneapolis, MN, United States

**Keywords:** muscle disease, therapeutic strategies, skeletal muscle, dystrophin, Duchenne muscular dystrophy, pathophysiology

## Abstract

Duchenne muscular dystrophy (DMD) is a severe, progressive, and ultimately fatal disease of skeletal muscle wasting, respiratory insufficiency, and cardiomyopathy. The identification of the dystrophin gene as central to DMD pathogenesis has led to the understanding of the muscle membrane and the proteins involved in membrane stability as the focal point of the disease. The lessons learned from decades of research in human genetics, biochemistry, and physiology have culminated in establishing the myriad functionalities of dystrophin in striated muscle biology. Here, we review the pathophysiological basis of DMD and discuss recent progress toward the development of therapeutic strategies for DMD that are currently close to or are in human clinical trials. The first section of the review focuses on DMD and the mechanisms contributing to membrane instability, inflammation, and fibrosis. The second section discusses therapeutic strategies currently used to treat DMD. This includes a focus on outlining the strengths and limitations of approaches directed at correcting the genetic defect through dystrophin gene replacement, modification, repair, and/or a range of dystrophin-independent approaches. The final section highlights the different therapeutic strategies for DMD currently in clinical trials.

## 1 Genetic basis and clinical presentation of DMD

Duchenne muscular dystrophy (DMD) is a severe X-linked recessive disorder caused by mutations in the dystrophin gene and consequent complete loss of dystrophin protein expression (Hoffman et al., 1987). The incidence of DMD is estimated at 1:5,000 boys worldwide, making it one of the most common recessive disorders in humans. The DMD gene that encodes dystrophin is located at position Xp21.1 of the X chromosome. DMD is the largest known gene in the human body, spanning 2.5 MB of DNA with 79 exons and 78 introns (Cohn and Campbell, 2000). The corresponding 14 kb mRNA transcript generates a 427 kDa protein with 3,685 amino acids (Hoffman et al., 1987; Emery, 2002).

Due to its massive size, the mutation rate for the dystrophin gene is quite high, with about one-third of mutations occurring *de novo* and the remaining caused by germline mosaicism or inheritance from a carrier mother (Nallamilli et al., 2021). More than 7,000 different dystrophin mutations have been identified in patients with DMD or Becker muscular dystrophy (BMD) (Bladen et al., 2015). Approximately 60% of mutations in patients with DMD are deletions, and 11% are duplications, with the remainder being small mutations affecting the coding sequence and splice sites (Bladen et al., 2015). Deletions and duplications occur predominantly in two hotspots of the DMD gene, located at exons 3–9 and 45–55 (Min et al., 2019a). The high mutation rate, along with the many different ways in which a frameshift could occur, raises significant challenges for the development of gene correction therapies applicable to patients with different dystrophin mutations.

DMD mutations preventing the production of dystrophin hold dire consequences for protection against muscle membrane stress. Dystrophin forms a mechanical link between cytoskeletal actin and the extracellular matrix (ECM), which protects the sarcolemma from the force associated with stretch and contraction (Campbell and Kahl, 1989; Boland et al., 1996; Houang et al., 2018). Secondary to deficient membrane stability is the disorganization of numerous proteins associated with dystrophin, as well as impairment of their distribution and cell signaling, both leading to severe and progressive muscle wasting (Emery, 1993; Jungbluth et al., 2018). Women act as carriers, and although affected men appear normal at birth, steady muscle weakness and wasting are first apparent in proximal limb muscles before spreading to more distal muscles (Mercuri et al., 2019), leading to the disease being typically diagnosed in the first few years of life (Tyler, 2003; Birnkrant et al., 2018). The earliest symptoms such as a waddling gait, frequent falls, difficulties standing up from a sitting position (Gower’s sign), and struggling to climb stairs can be seen between ages 2 and 3 (Emery, 2002). Affected children exhibit delayed gross motor development, and at ages 10–12, most patients require a wheelchair (Tsuda, 2018). As muscle weakness progresses, scoliosis and joint contractures develop, which promote restrictive lung disease (Bushby et al., 2010). Assisted ventilation is necessary by about 15–20 years of age, and the majority of DMD patients die from cardiac/respiratory failure between 20 and 30 years of age, even when optimal care has been administered (Mendell et al., 2012; Mercuri et al., 2019). Additionally, the corresponding immobility hastens the decline in bone density and increases the risk of fracture (McDonald et al., 2002).

The cardiac muscle must withstand significant mechanical stress to pump blood throughout the body, and the consequences of this stress are greatly exacerbated in DMD patients. Membrane instability leads to gradual cardiac myocyte death, resulting in fibrosis and secondary fatty infiltration (Townsend et al., 2011; Houang et al., 2018; Tsuda, 2018). This process decreases diastolic/systolic function, which plays a role in the atrophy and remodeling that gives rise to heterogeneous pathological alterations to the ventricular myocardium (Amin et al., 2002; Desguerre et al., 2009; Spurney, 2011a; Lee et al., 2014). Life-threatening consequences of cardiac muscle dystrophin deficiency include dilated cardiomyopathy, arrhythmias, and congestive heart failure (Kamdar and Garry, 2016; McNally and Wyatt, 2017; Buddhe et al., 2019; Meyers and Townsend, 2019). Along with these debilitating effects on cardiac function, the pulmonary ramifications of DMD are a key co-contributor to patient mortality (Emery, 2002). Weakened respiratory muscles, particularly the diminishment of diaphragm function, cause severe ventilatory insufficiency (Benditt and Boitano, 2005; Bushby et al., 2010). Additionally, incidences of otherwise benign upper respiratory tract infections can lead to serious acute respiratory failure in DMD, as patients cannot cough effectively. Diminished cough capacity causes airway secretions to be retained that in turn increases the risk of pneumonia and atelectasis (Bushby et al., 2010). Reduction in vital capacity and elevated residual volume due to impaired expiratory muscle function is also exhibited, which further necessitates assisted ventilation later in the disease progression (Bach et al., 1997; Benditt and Boitano, 2005; Bushby et al., 2010).

Although dystrophin deficiency in muscle tissues is the primary cause of the life-limiting issues associated with DMD, a lack of brain dystrophin has consequences as well (Duan et al., 2021). DMD patients reportedly have a higher susceptibility to seizures as compared to dystrophin replete individuals. Additionally, cognitive, neuropsychological, and neurobehavioral abnormalities are more likely to occur in DMD patients (Boyce et al., 1991; Kim et al., 1995). The general intelligence of affected boys is one standard deviation below the normal mean IQ, with 34.8% of patients reported to have intellectual disabilities (Cotton et al., 2001). Moreover, ADHD, autism spectrum disorder, and moderate-to-severe reading problems were exhibited in 11.7%, 3.1%, and 20% of patients, respectively (Hendriksen and Vles, 2006; Hendriksen and Vles, 2008).

The survival of patients with DMD has improved over time. With optimal care, patients with DMD can survive to live in their thirties and forties, mainly owing to the development of guidelines for care and management and improved treatment for cardiopulmonary dysfunction (Birnkrant et al., 2018).

## 2 Dystrophin function

Dystrophin is an essential cytoskeletal protein in muscle that localizes to the inner surface of the muscle cell membrane and is enriched at the costameres and sites of cell–cell contact (Ervasti and Campbell, 1993). Dystrophin is the connection point joining the actin cytoskeleton to the inner surface of the sarcolemma (Hoffman et al., 1987; Ahn and Kunkel, 1993). Dystrophin has the characteristic biochemical properties of a membrane cytoskeletal protein and is recognized as a major member of the spectrin-type superfamily of actin-binding proteins (Petrof et al., 1993; Straub et al., 1997). In muscle cells, dystrophin plays an important role in maintaining membrane integrity and preventing membrane rupture (Emery, 2002; Gao and McNally, 2015).

The dystrophin protein has four main functional domains: 1) an N-terminal region with homologies to the actin-binding domains of α-actinin that anchors the protein to the cytoskeleton; 2) a central rod domain containing 24 spectrin-like repeats separated by four hinge regions; 3) a cysteine-rich domain that anchors dystrophin to the muscle membrane via interaction with the transmembrane protein β-dystroglycan; and 4) a C-terminal domain that binds dystrobrevin and syntrophins, mediating sarcolemma localization (Ervasti, 2013). The N-terminal domain includes two calponin homology domains (CH1 and CH2). This conventional CH-actin binding domain binds directly to F actin, anchoring dystrophin to the cytoskeleton (Rybakova et al., 1996). The spectrin-like repeats in the central rod of dystrophin indicate that dystrophin works as a shock absorber, helping to resist repeated rounds of muscle contraction and relaxation. The rod domain also harbors a second actin-binding motif (ABD2). ABD2 is found near the middle of the rod and works in concert with ABD1 to form a strong lateral association with actin filaments (Straub et al., 1992). The rod domain also mediates dystrophin interaction with microtubules via spectrin-like repeats 20–23 and is required for the organization of the microtubule lattice in skeletal muscle cells. Furthermore, the rod forms a flexible linker between the amino- and carboxy-termini. Four short proline-rich spacers, called “hinges,” are interspersed in the mid-rod domain providing elasticity to the protein (Koenig et al., 1988). Hinge 4 is at the end of the rod domain and contains a WW domain, a domain implicated in protein–protein interactions (Koenig and Kunkel, 1990; Straub et al., 1992). The WW domain, along with two neighboring EF-hands, binds the carboxy-terminus of β-dystroglycan, anchoring dystrophin at sarcolemma (Koenig et al., 1988). In dystrophin, the two EF-hands are located in the cysteine-rich domain, which resides between the central rod and the C-terminus. The cysteine-rich domain also contains a zinc finger (ZZ) domain (Ishikawa-Sakurai et al., 2004). The carboxy-terminal (CT) domain is α-helical and provides binding sites for dystrobrevin and syntrophins, mediating their sarcolemma localization.

Connection is achieved through the dystrophin-glycoprotein complex (DGC), which consists of extracellular (α-dystroglycan), transmembrane (sarcoglycans, β-dystroglycan, sarcospan), and cytoplasmic proteins (dystrophin, syntrophins, neuronal nitric oxide synthase, dystrobrevin), providing a link between the intracellular cytoskeleton and the ECM (Campbell and Kahl, 1989; Ervasti and Campbell, 1991).

It has been shown that α-dystroglycan resides on the extracellular surface of the sarcolemma and functions as a receptor for extracellular ligands such as laminin (Montanaro et al., 1999). Here, α-dystroglycan is tightly associated with β-dystroglycan, a transmembrane protein that also interacts with dystrophin. At the sarcolemma, β-dystroglycan is tightly associated with the sarcoglycan subcomplex, which is composed of four single-pass transmembrane proteins: α-sarcoglycan, β-sarcoglycan, γ-sarcoglycan, and δ-sarcoglycan (Ervasti and Campbell, 1993). The sarcoglycan complex is also tightly associated with sarcospan, a small transmembrane protein. On the cytoplasmic side of the sarcolemma, dystrophin maintains its membrane localization by interacting with β-dystroglycan. Dystrophin binds to the intracellular actin network to link the cytoskeleton to DGC, which in turn connects to basal lamina by interacting with ECM ligands. Other cytoplasmic components of DGC include α-dystrobrevin, syntrophins, and neuronal nitric oxide synthase (nNOS) (Ervasti, 2013). The α-dystrobrevin/syntrophin triplet associates with dystrophin and anchors nNOS to the sarcolemma (Guiraud et al., 2015).

## 3 Pathophysiological mechanisms of Duchenne muscular dystrophy

### 3.1 Membrane instability

In striated muscle, force is generated through cycles of contraction and relaxation of muscle fibers as driven via the sarcomere (Gordon et al., 2000). As discussed previously, in healthy muscle, the integrity of the sarcolemma is maintained through interactions between the sarcolemma, cytoskeleton, and ECM through the DGC and the integrin complex (Houang et al., 2018; Duan et al., 2021). However, in DMD, due to the lack of dystrophin and its associated proteins, the physical connection between the sarcolemma, cytoskeleton, and ECM is lost, causing the sarcolemma to become leaky and highly susceptible to injury (Guiraud et al., 2015; Houang et al., 2018; Duan et al., 2021). This mechanism is supported by several observations. Previous work has identified hallmarks of muscle injury in DMD patients in the form of sarcolemmal tears (so-called “delta” lesions) (Mokri and Engel, 1975; Pestronk et al., 1982; Duan et al., 2021), increased levels of creatine kinase in the serum, and the uptake of large proteins including albumin and Evans blue dye (Houang et al., 2018; Duan et al., 2021). As levels of muscle damage are directly linked to levels of muscle stress, those muscles that undergo higher levels of stress are more likely to incur higher levels of damage. This can be seen in skeletal muscles involved in the exercise, specifically those muscles undergoing lengthening contractions, where it has been shown that repetitive contractions lead to increases in sarcolemmal damage (Petrof et al., 1993; Allen et al., 2016). This can also be seen in the diaphragm, where increased levels of injury are seen in DMD patients (Stedman et al., 1991). Taken together, these works demonstrate the need for the dystrophin complex to maintain the structural integrity of the muscle sarcolemma and highlight the devastating consequences of muscle fiber membrane stability in DMD.

### 3.2 Calcium dysregulation

A major consequence of the increased levels of membrane instability and leakiness in DMD muscles is an increase in intracellular calcium levels and disrupted calcium homeostasis within the muscle (Hopf et al., 1996; Mareedu et al., 2021). There is some debate over the mechanisms leading to increased intracellular calcium in the DMD muscle. Previous work has suggested that calcium can enter the muscle fibers through membrane tears (Yasuda et al., 2005; Houang et al., 2018), through a number of different calcium channels, including store-operated, voltage-gated, or stretch-activated (Mareedu et al., 2021), or through sodium channels (Mareedu et al., 2021).

Although the mechanism of calcium entry is not fully resolved, it is clear that increased calcium levels in DMD muscles have a significant negative impact on function. This can be seen through the myriad of calcium-signaling pathways in the cell, including calmodulin (Pertille et al., 2010), calpains (Gailly et al., 2007), and dysferlin (Bansal et al., 2003; Houang et al., 2018), all of which can negatively impact calcium cycling and muscle repair. Another possible mechanism of calcium dysfunction in DMD that has become a recent area of focus is the sarcoplasmic reticulum (SR) (Duan et al., 2021). In DMD muscle fibers, it has been shown that there is an increase in passive calcium leak from the SR, and therefore sarco-endoplasmic reticulum calcium ATPase (SERCA) function is reduced, leading to an increase in cytosolic calcium levels (Duan et al., 2021; Mareedu et al., 2021). It has been suggested that this process may occur through SR crosstalk with reactive oxygen species (ROS) (Papa and Skulachev, 1997; Houang et al., 2018; Mareedu et al., 2021). Further damage in the muscle can also be the result of calcium-mediated changes in mitochondrial function, which can lead to a reduction in ATP production and loss of membrane potential (Kyrychenko et al., 2015; Houang et al., 2018; Kang et al., 2018; Mareedu et al., 2021). Both of these mechanisms are discussed in more detail as follows. It is evident in dystrophic muscle fibers that the number of ways calcium-signaling can negatively impact muscle function and the increased levels of cytosolic calcium in DMD muscle is unmistakably a driving force in the pathology of the disease.

### 3.3 Inflammation

Immune cell infiltration is a main feature of DMD and is strongly associated with disease severity (Rosenberg et al., 2015; Villalta et al., 2015; Saclier et al., 2021). In DMD patients, inflammation onset precedes the onset of clinical symptoms (Pescatori et al., 2007). The involvement of the immune system after DMD onset, when there is already marked muscle damage, is well established (Vidal et al., 2008; Burzyn et al., 2013; Mojumdar et al., 2014; Mázala et al., 2020; Saclier et al., 2021). However, little is known about the role of inflammation in the earliest phase of DMD prior to the clinical onset (Evans et al., 2009).

Skeletal muscle is host to a heterogeneous combination of endothelial, stem, and immune cells, including macrophages, natural killer (NK) cells, T cells, B cells, and neutrophils (Tidball and Villalta, 2010). Immune cells play an important role in skeletal muscle homeostasis, repair, and regeneration (Tidball and Villalta, 2010; Castiglioni et al., 2015; Sciorati et al., 2016). The unique immune-privileged status of skeletal muscle (Sciorati et al., 2016) relies on the absence of constitutive MHCI expression and the lower number of antigen-presenting cells and pro-inflammatory cells in homeostasis, resulting in less necrosis and a lower capacity to generate abscesses (Evans et al., 2009; Tibdall et al., 2018). In the setting of acute muscle injury, neutrophils and pro-inflammatory macrophages are early players and are essential in clearing damaged fibers. They are then gradually replaced by anti-inflammatory pro-regenerative macrophages to stimulate fibers regeneration and fibrosis (Evans et al., 2009; Tidball and Villalta, 2010; Castiglioni et al., 2015; Tidball et al., 2018; Gehlert and Jacko, 2019). A distinct population of regulatory T cells (Foxp3+ Treg) drives the transition to a pro-regenerative state and potentiates muscle regeneration and repair (Burzyn et al., 2013).

As detailed previously, membrane instability is a primary defect in DMD. In healthy muscles, intense exercise causes membrane damage and leakage of cytoplasmic content into the extracellular compartment (Sorichter et al., 1998; Sorichter et al., 1999). This is associated with increased circulating levels of muscle proteins such as fatty acid binding protein and muscle-specific creatine kinase that can be used as biomarkers to detect and evaluate these injuries (Sorichter et al., 1998). Due to the muscle’s unique immune privilege, rapid membrane repair mechanisms, and membrane stability conferred by intact dystrophin, this inflammatory response is limited, controlled, and quickly resolved in healthy muscle (Sciorati et al., 2016). In DMD, the muscle loses its immune privilege. Membrane instability caused by dystrophin deficiency leads to the continuous release of cytoplasmic content, in particular, damage-associated molecular patterns (DAMPs) that are ligands to toll-like receptors (TLRs), purinoceptors (P2RX7), and other pattern recognition receptors (PRRs) on muscle and immune cells (Henriques-Pons et al., 2014; Giordano et al., 2015; Sinadinos et al., 2015). Upon activation, PRR downstream signaling cascades initiate the innate immune response. Moreover, pro-inflammatory cytokines induce the expression of MHCI and MHCII on muscle fibers, thus breaking down the muscle immune privilege. In addition to membrane instability, intracellular calcium overload and increased oxidative stress further induce tissue damage, contributing to triggering the inflammatory cascade in dystrophin-deficient muscles (Wehling et al., 2001; Henriques-Pons et al., 2014).

The immune cell infiltration in young DMD patients is predominantly formed by macrophages and T cells (Evans et al., 2009). Similar findings were revealed in dystrophin-deficient murine muscles (Lozanoska-Ochser et al., 2018). Early in the DMD time course, degenerated muscle fibers are cleared and repaired by regeneration (Tidball and Villalta, 2010; Tidball et al., 2018). The co-existence of lesions of different ages and the overlapping of pro-inflammatory and anti-inflammatory signaling prevent the full resolution of inflammation and sustain a chronic inflammatory response, which further progresses muscle damage. As the disease progresses, the impaired regenerative capacity and the chronic inflammatory environment of dystrophic muscle lead to fibrosis and adipose tissue accumulation (Vidal et al., 2008; Evans et al., 2009; Rosenberg et al., 2015; Mázala et al., 2020).

Macrophages play a key role in the disease progression of DMD (Rosenberg et al., 2015). Genetic ablation of CCR2 in a dystrophin-deficient mouse model (mdx), which prevents the monocytes from exiting the bone marrow, reduces the pro-inflammatory macrophage infiltration into the dystrophin-deficient muscle and is shown to improve muscle histology and function (Mojumdar et al., 2014). Bhattarai et al. (2022) showed, in the necrotic phase, a TLR4-dependent alteration of the inflammatory phenotype of the mdx mice bone-marrow-derived macrophages, with implications in major remodeling of the macrophage metabolic and epigenetic landscape. These changes correspond to characteristics of trained immunity and indicate that trained immunity can play an important role in DMD pathogenesis and progression (Bhattarai et al., 2022). T cells also play a crucial role in DMD pathogenesis (Lozanoska-Ochser et al., 2018; Tidball et al., 2018). CD4^+^ helper T cells promote the immune response by producing inflammatory cytokines (Spencer et al., 2001). CD8^+^ T-cell perforin-mediated cytotoxicity and fatty acid synthetase-induced cytotoxicity cause muscle fiber apoptosis (Spencer et al., 1997; Cai et al., 2000; Spencer et al., 2001). Effector T cells were recently shown to be one of the earliest cell types infiltrating the dystrophic skeletal muscle (in 2-week-old mdx mice) via a protein kinase C θ- (PKCθ-) dependent mechanism (Lozanoska-Ochser et al., 2018). The absence of PKCθ, a key regulator of effector T-cell activation, significantly reduced T-cell infiltration and the innate immune cell infiltrate in mdx/Prkcq^−/−^ muscle (mdx lacking Prkcq, the PKCθ-coding gene), and improved the muscle phenotype (Lozanoska-Ochser et al., 2018).

### 3.4 Mitochondrial energetics

Mitochondria play a pivotal role in the bioenergetics of muscle fibers via oxidative phosphorylation. The electron transport chain oxidizes NADH or FADH_2_ through Complexes I and II in the matrix of the mitochondria and generates a proton gradient across the inner membrane of the mitochondria while transporting the electrons (Ahmad et al., 2022). The ATP synthase utilizes this proton gradient to synthesize ATP from ADP and inorganic phosphate. Fine orchestration of the electron transport chain complexes is essential for normal bioenergetics. DMD results in an abnormal environment that is not favorable for mitochondrial homeostasis (Cole et al., 2002; Allen et al., 2016). Instability in the DMD muscle membrane triggers extracellular Ca^2+^ inflow, leading to mitochondrial Ca^2+^ overload (Turner et al., 1988; Mareedu et al., 2021; Garcia-Castaneda et al., 2022). Although Ca^2+^ buffering is another key function of the mitochondria in the muscle through the mitochondrial Ca^2+^ uniporter (MCU), pathogenically high levels of cytoplasmic [Ca^2+^] can lead to Ca^2+^ overload in the mitochondria and trigger the opening of the mitochondrial permeability transition pore (mPTP) (Hurst et al., 2017; Briston et al., 2019).

Morphologically, mitochondria in dystrophin-deficient muscle display decreased size and sparse cristae formation, which are attributed to an increased mitochondrial fission/fusion cycle (Pant et al., 2015; Kang et al., 2018; Moore et al., 2020). Furthermore, diminished protein expression involved in PINK1/PARKIN mitophagy also causes structural damage and mitochondrial dysfunction in dystrophic cardiac muscles (Pant et al., 2015). These observations combined suggest that increased mitochondrial dynamics and suppressed mitophagy in dystrophin-deficient muscle also contribute to mitochondrial dysfunction (Pant et al., 2015).

ATP synthesis capacity has also been found to be disrupted in dystrophin-deficient muscle, and this is shown by a reduction in mitochondrial density and maximal ATP synthesis rate (Percival et al., 2013), as well as mitochondrial respiration (Passaquin et al., 2002; Onopiuk et al., 2009; Schuh et al., 2012). Interestingly, mitochondrial dysfunction and metabolic abnormalities were found to precede the onset of myofiber necrosis in young mdx mice (Townsend et al., 2011; Moore et al., 2020).

### 3.5 Reactive oxygen species dysregulation

In healthy muscle, a basal level of reactive oxygen species (ROS) is produced, and this is required for optimal cellular function, such as contraction (Reid et al., 1992; Reid, 2001; Powers et al., 2011). Muscles from animal models and patients with DMD produce significantly more free radicals than normal muscle, making the dystrophic muscle more vulnerable than healthy muscle to oxidative stress. Electrons leaked from the electron transport chain in mitochondria are one of the major sources of ROS production in muscle fiber cells (Fridovich, 2004). Specifically, electrons leaked from Complexes I and III combine with oxygen to form superoxide and eventually hydrogen peroxide by superoxide dismutase (e^−^ + O_2_ → O_2_^−^ → H_2_O_2_) at the mitochondrial matrix and cytosol (Chance et al., 1979). Within a healthy cell, ROS and antioxidants maintain a delicate balance, referred to as the optimal redox state. However, an uncontrolled amount of ROS production in pathology that exceeds the capacity of innate antioxidants generates redox unbalance leading to pathological dysfunction and even cell death (Powers et al., 2011).

Within the muscle fiber, mitochondria form a reticular network that surrounds the contractile apparatus, providing a short distance for the ATP supply within the healthy muscle (Glancy et al., 2015). In pathology, however, this proximity becomes deleterious for the redox-sensitive contractile proteins. Myofilament structures, including troponin, tropomyosin, myosin, and actin, contain a thiol group that is sensitive to redox balance, and exposure to an excessive amount of ROS disrupts myofilament Ca^2+^ sensitivity and cross-bridge kinetics causing muscle contraction dysfunction (Andrade et al., 1998; Terrill et al., 2013).

DMD is noted for pathologically elevated ROS production from the mitochondria in muscle. Specifically, Complex I-supported maximal H_2_O_2_ production has been found to be markedly elevated in skeletal muscle from young mdx mice (Godin et al., 2012; Hughes et al., 2019). Furthermore, skeletal muscles from mdx mice display decreased Ca^2+^ retention capacity, leading to susceptibility to Ca^2+^-induced mitochondrial permeability transition pore (mPTP) opening, as discussed previously. Interestingly, mdx skeletal and cardiac muscle have an adaptive elevation in ROS buffering capacity that counteracts the elevated ROS production from the mitochondria (Godin et al., 2012; Hughes et al., 2019; Hughes et al., 2020), and although this can temporarily dampen ROS, it still leads to an increase in oxidative damage as the disease progresses. Chronic inflammation due to sarcolemma damage and myofiber necrosis in dystrophin-deficient cardiac and skeletal muscle is a central feature of DMD, and this contributes to elevated ROS production from mitochondria (Terrill et al., 2016; Tulangekar and Sztal, 2021). Lastly, pathological conditions, including DMD, that impact mitochondrial function can cause more severe effects on the diaphragm than on limb muscle due to the relatively greater ROS emission rates per mass and mitochondrial content (Hahn et al., 2019). This suggests that the mitochondria as a crucial therapeutic target when considering the involvement of diaphragm weakness and wasting leads to cardiorespiratory failure in DMD patients (Mead et al., 2014).

NADPH oxidase 2 (NOX2) also has a considerable role in the pathology of DMD muscle as a source of ROS (Williams and Allen, 2007; Jung et al., 2008; Whitehead et al., 2010). NOX2 is activated by the microtubule-associated protein Rac1 upon mechanical stretching of the muscle, leading to ROS production. This process is greatly increased in DMD as the microtubule lattice becomes disorganized and dense due to the lack of dystrophin-microtubule interaction (Khairallah et al., 2012). NOX2 in phagocytic cells produces superoxide during respiratory bursts for host immunity against bacteria and infections. In DMD, continuous contraction-induced muscle membrane damage also leads to NOX2 activation (Moe et al., 2011; Zhao et al., 2015). Furthermore, biopsies from DMD patients showed greater gene expression of NOX2 compared to age-matched controls (Petrillo et al., 2017), and inhibition of NOX2 expression improved muscle function in mdx mice (Whitehead et al., 2015). Another important source of free radicals in dystrophin-deficient muscles is reactive nitrogen species produced by the cytosolic activation of delocalized nNOS. Together, this free-radical production leads to increased DNA, protein, and lipid oxidation in dystrophin-deficient muscles (Kim et al., 2013; Grounds et al., 2020). Mitochondria and NOX2 have shown potential targets for ROS production.

Indeed, there have been several reports showing significant improvements in muscle function, as well as prevention of myofiber necrosis and membrane permeability from mdx skeletal muscle by N-acetylcysteine supplementation (Whitehead et al., 2008; Terrill et al., 2012; Pinniger et al., 2017).

### 3.6 Fibrosis

Fibrosis is a wound-healing process wherein an excessive amount of connective tissue is formed, leading to permanent scar tissue. The fraction of ECM in normal muscle is ∼5%, which can be drastically increased in diseased or injured muscles (Lieber and Ward, 2013). Repeated injury/regeneration cycles and chronic inflammation cause fibrosis and loss of function, and this is another phenotypic feature of DMD (Kharraz et al., 2014; Gallardo et al., 2021). In DMD, fibrosis decreases contractile function and reduces the amount of healthy muscle for therapy (Kharraz et al., 2014). Muscle membrane damage in DMD and the subsequent accumulation of acidic metabolites and inflammation amplification initiate fibrotic tissue production by extracellular fluid containing fibronectin, glycosaminoglycans, and proteoglycans (Klingler et al., 2012). Specifically, transforming growth factor-β (TGF-β) is released from the skeletal muscle in injury or inflammation, and this activates myofibroblasts, leading to excessive production of ECM dominated by type I and type III collagen (Zhou et al., 2006; Lieber and Ward, 2013; Meng et al., 2016). Myostatin, which is a negative regulator of muscle mass, also stimulates muscle fibroblasts to proliferate ECM proteins (Li et al., 2008). In addition, angiotensin II, collagen triple helix repeat-containing 1 (Cthrc1) protein, and Wnt signaling regulate fibrogenic turnovers of the muscle cells (Bedair et al., 2008; Li et al., 2011; Cisternas et al., 2014).

The ECM functions as the major source of passive load-bearing in muscles. When comparing the normalized stiffness of a single muscle fiber, fiber bundle (ECM intact), and fiber alone (ECM non-intact), muscle fiber bundles had much higher stress, and this demonstrated that the ECM provides a large portion of the load-bearing function (Meyer and Lieber, 2011). In this light, DMD skeletal muscle displays significantly greater stiffness (Lacourpaille et al., 2015; Lacourpaille et al., 2017; Yu et al., 2022). In animal models of DMD, the lower limbs of mdx mice displayed an increase in the amount of collagen, and this was reported to have a close relationship with increased stiffness in the muscle (Brashear et al., 2021).

Because the fibrotic process begins early in DMD, therapeutic approaches aiming to reduce fibrosis have gained increased interest as a potential strategy for DMD. Therapeutic approaches inhibiting fibrosis (collagen type 1) have been shown to increase the expression of utrophin and the number of revertant myofibers (Levi et al., 2015). Other therapeutic targets that have shown the ability to limit the accumulation of fibrosis in DMD models and in patients include the renin-angiotensin system, cytokines, and tyrosine kinase receptors (Kharraz et al., 2014), ACE inhibitors, ARBs, and mineralocorticoid receptor antagonists (Heier et al., 2019; Kim et al., 2019).

## 4 Therapeutic strategies for DMD

Despite major therapeutic advances over the past 30 years, there is no curative treatment for DMD (McNally and Pytel, 2007; Mercuri et al., 2019). Nonetheless, a multidisciplinary medical, surgical, and rehabilitative approach targeting the symptoms of DMD can alter the natural course of the disease, improving longevity and quality of life. Glucocorticosteroids, such as deflazacort or prednisone, are the current standard of care and the most widely used treatment for DMD patients (Bushby et al., 2004). The exact mechanism by which glucocorticosteroids delay disease progression in DMD is not fully understood. However, it has been suggested that glucocorticosteroids increase total muscle mass and strength in patients with DMD through the stimulation of insulin-like growth factors, decreased inflammation, decreased lymphocyte reaction and cytokine production, and enhanced myoblast proliferation (for more information on this topic, please see earlier reviews (Angelini and Peterle, 2012; McDonald et al., 2018; McDonald et al., 2020). Although glucocorticosteroids and many other pharmacological strategies can delay symptoms by targeting the secondary effects of DMD, many are only partially effective because they treat just one aspect of DMD pathogenesis. Nevertheless, long-term corticosteroid treatment has a variety of unfavorable side effects, including weight gain, short stature, puberty delay, behavioral issues, and pathologic bone fractures (Connolly et al., 2002). A promising oral glucocorticosteroid analog named VBP-15 was recently shown to improve muscle strength without side effects in the mdx mouse (Damsker et al., 2013; Heier et al., 2013), and a phase 1 clinical trial is currently underway on healthy human volunteers.

As reviewed before, there has been great interest in developing genetic approaches to treat DMD. Herein, we highlight some of the most promising therapeutic strategies that have been used to treat DMD, including gene therapy approaches via direct replacement of dystrophin and newer approaches involving the manipulation of the cellular machinery at the level of gene transcription, mRNA processing, or translation.

### 4.1 Microdystrophin viral gene therapy

Viral-mediated gene therapy has long been considered a potential therapeutic strategy to treat DMD based on its capability to restore the missing dystrophin by providing a functional copy of the dystrophin gene or repairing dystrophin, thereby restoring dystrophin throughout the body. The fact that DMD patients can be candidates for replacement gene therapy regardless of the underlying dystrophin mutation makes this a very attractive approach to correct DMD defects (Gregorevic et al., 2004; Chamberlain and Chamberlain, 2017; Duan, 2018a; Crudele and Chamberlain, 2019).

Implementing DMD gene therapy has turned out to be a very challenging endeavor, involving two main challenges. The first problem is the dystrophin gene’s immense size. The dystrophin gene, including its coding sequence, poses a problem for viral vector packaging (Roberts et al., 1993). The second hurdle involves systemic delivery to treat all the striated muscles in the body. Thus, a whole-body systemic therapy approach is required (Townsend et al., 2007; Chamberlain and Chamberlain, 2017). Numerous truncated dystrophin designs and body-wide systemic viral gene transfer approaches have been investigated in many labs to overcome these barriers (Gregorevic et al., 2004; Odom et al., 2007; Duan, 2016a; Duan, 2018b; Ramos et al., 2019).

The most common viral vectors implemented for neuromuscular disorders are lentiviruses and adeno-associated viruses (AAV). Table 1 provides a comparison of different vector systems for treating DMD. Among all the virus-based technologies in use for DMD clinical trials, adeno-associated viral (AAV) vectors encoding mini- or microdystrophins show encouraging treatment results in the preclinical setting (Kawecka et al., 2015; Duan, 2018b). The full-length dystrophin cDNA is far larger than the 4.7 kb packaging capacity of AAVs. Hence, these vectors are unable to deliver the full-length dystrophin cDNA. Two or three AAV vector (tri-AAV) *in vivo* recombination systems have been reported to deliver full-length dystrophin, albeit at very low efficiency (Lostal et al., 2014; Morgan and Muntoni, 2021). Studies have been directed to generate engineered AAVs with increased packaging capacity that induces efficient transduction associated with a modest immune response (Rogers et al., 2011). The currently used recombinant AAVs (rAAV) are erased of all viral genes, specifically the rep and cap genes that encode the nonstructural and structural proteins, and replaced by a sequence of interest, resulting in a maximum encapsidation capacity of around 5 kb (Dong et al., 1996). These vectors mostly remain episomal as extrachromosomal concatemers and rarely integrate into the genome. However, Schnepp et al. (2003) reported rAAV integration rates of about 0.5% in muscles from adult mice. AAV serotypes display a variety of cellular tropisms with a range of efficiency for targeting different muscles (Gao et al., 2004; Zincarelli et al., 2008).

**TABLE 1:** Viral vector comparison in terms of their applicability to DMD gene therapy.

Characteristics	Adenovirus	Lentivirus	AAV
Genome	dsDNA	ssRNA	ssDNA
Capacity (kb)	Up to 30 kb	Up to 10 kb	Up to 4.7 kb
Genome integration	NO	Yes	On rare events
Expression duration	Transient	Persistent	Intermediate
Non-dividing cell transduction	Yes	Yes	Yes
Ability to target satellite cells	NO	Yes	NO
Immune response	High	Low	Very low
Risk	Elicits viral-induced inflammation/cytotoxicity	Insertional mutagenesis/potential for replication of competent virus	High safety profile

Initial studies featuring direct AAV vector injection into muscle showed high-level and durable gene transfer (Kessler et al., 1996; Xiao et al., 1996), although this was limited to the injected muscles and represents a non-clinically feasible condition as a continuous injection would be necessary for a therapeutic treatment modality. A major advancement was achieved when the Chamberlain lab (Gregorevic et al., 2004) and Xiao lab (Wang et al., 2005) demonstrated the success of using effective systemic gene transfer to cause whole-body muscle transduction in rodents with AAV pseudotype-6 vectors. Subsequently, the Duan lab reported the success of using systemic AAV-9 transduction in canines (Yue et al., 2008). Currently, a broad variety of AAV capsids that are either isolated from nature or engineered in laboratories have been used to accomplish systemic delivery (Townsend et al., 2007; Kotterman and Schaffer, 2014; Duan, 2016b). For instance, in rodents and large animals, a single intravenous administration of AAV-6, AAV-8, and AAV-9 led to whole-body muscle transduction. Additionally, limiting the expression to tissues of interest can be achieved by utilizing cell type-specific promoters. These advances spurred the development of AAV as the vector of choice for muscle gene therapy (Salva et al., 2007; Wang et al., 2014; Duan, 2016b).

Because AAV vectors rarely integrate into the muscle fiber genome, it follows that with muscle turnover, the microdystrophin transgenes will be diluted and lose effectiveness over time. As AAV vectors do not effectively target satellite cells, upon muscle injury, the satellite cells that are recruited to take part in the repair process will not contain the therapeutic transgene, causing the transduced cells to become diluted and eventually disappear with time (Arnett et al., 2014).

AAV administration may also induce an innate or cellular immune response to the vector, including anti-AAV capsid-neutralizing antibody production. In addition, some patients already have pre-existing AAV-neutralizing antibodies, which exclude them from receiving this treatment (Calcedo et al., 2009; Boutin et al., 2010). Strategies to counter this antibody response, such as by plasmapheresis, alternative AAV serotype, or immune-modulating drugs, are being investigated. The high safety profile of AAVs is evident by the fact that the most severe side effect to date using various AAV serotypes in several clinical trials was a temporary tissue inflammation that did not result in any long-term damage (Büning and Schmidt, 2015). Several clinical trials are investigating the safety and tolerability of this promising therapy in DMD patients (for more details, see *Clinical trials* section).

As clinical trials continue to develop, other factors controlling the function and stability of the transgene are being evaluated. The Chamberlain group reported that AAV-mediated delivery of microdystrophin resulted in continued expression that was maintained for at least 27 months in both skeletal and cardiac muscles (Ramos et al., 2019). Conversely, Dupont et al. (2015) reported short-lived AAV-derived therapeutic mRNAs in the dystrophic milieu. This finding highlights the necessity for additional elements to be considered when rAAV-mediated gene therapy is being optimized. Designing improved gene therapy treatments for DMD will be made possible with continued research into vector development, optimization of expression cassettes, and further investigations, including immune modulation.

As previously indicated, the coding sequence of the muscle isoform of the dystrophin gene is approximately 11 kb long, and this length presents a significant difficulty in developing gene transfer therapy (Duan, 2018a). Observations in patients with BMD, typically a milder and less common form of muscular dystrophy, have suggested that the transfer of the full-length dystrophin open reading frame (ORF) may not be necessary for developing effective gene therapy. Thus, the disease phenotype may be alleviated with a smaller dystrophin gene construct. In BMD, mutations cause the loss of some exons but do not abolish dystrophin expression, as in the case of DMD (England et al., 1990).

The capacity to excise specific coding regions within the dystrophin gene while maximizing protein function has been made possible by the availability of the full-length sequence of dystrophin together with the knowledge of related protein domains (Jung et al., 1995; Rafael et al., 1996; Lai et al., 2009). Some domains of the dystrophin sequence are reported to be somewhat dispensable for protein function. Hence, a series of internally truncated dystrophin constructs lacking regions of the rod domain and/or the C-terminal domain were devised to be used in place of the full dystrophin (Yuasa et al., 1998; Harper et al., 2002; Goyenvalle et al., 2011; Chamberlain and Chamberlain, 2017). Microdystrophins are partially functional proteins that lack more than half of the typical amino acid sequence of dystrophin. Early versions of microdystrophin, which are about one-third the size of the human dystrophin cDNA (around 3.7 kb in size), lacked the C-terminal domain and up to 20 SRs. In pre-clinical studies, these were very successful at reducing necrosis in dystrophic mdx mouse muscles (Phelps et al., 1995; Gregorevic et al., 2006). They have also been successful in enhancing force generation in striated muscles of dystrophic mouse and canine models for DMD and in preserving the sarcolemma from the injury induced by contraction (Harper et al., 2002; Watchko et al., 2002; Abmayr and Chamberlain, 2006; Le Guiner et al., 2017). These characteristics are attained via micro-dys binding to γ-actin filaments and β-dystroglycan through the N-terminal actin-binding domain and the cystine-rich β-dystroglycan binding domain, respectively. Thus, microdystrophin can form a mechanically robust connection between the subsarcolemmal cytoskeleton and the ECM (Corrado et al., 1996; Rafael et al., 1996; Harper et al., 2002; Gardner et al., 2006; Ervasti, 2013).

**FIGURE 1:**
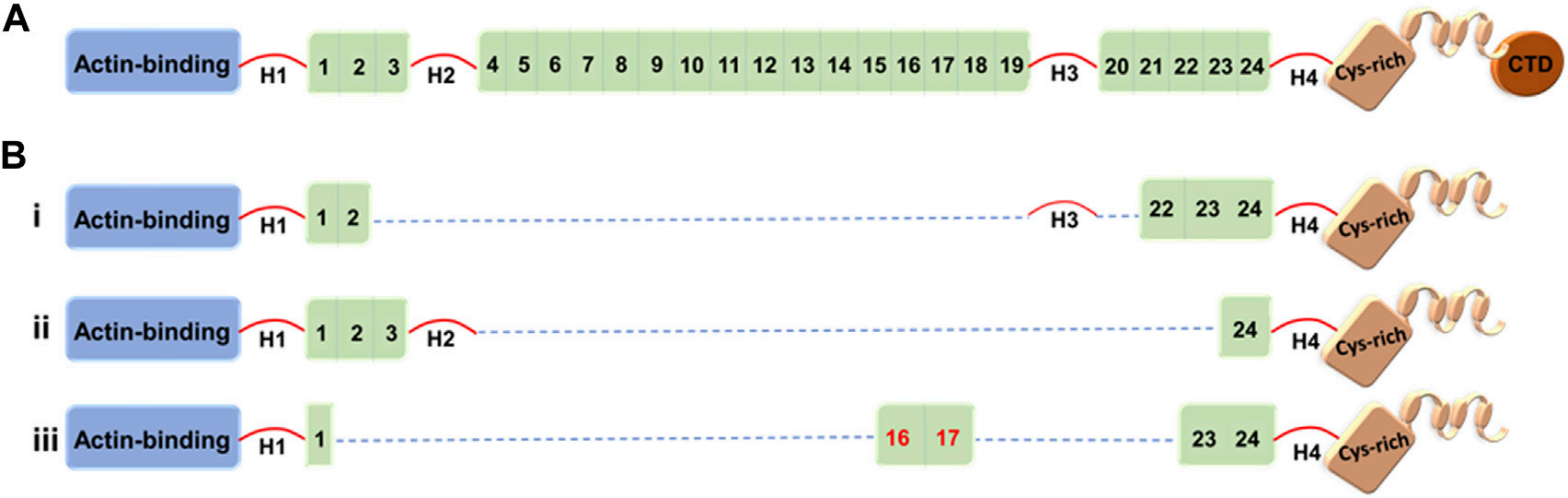
Full-length dystrophin and representative truncated dystrophins currently in use in clinical trials. **(A)** Full-length dystrophin *versus* **(B)** the truncated dystrophin constructs: i) mini-dystrophin Δ3990, ii) microdystrophin ΔR4-23/ΔCT (µDysH2) and iii) microdystrophin μDys-5R. Domains are identified as the N-terminal actin-binding domain, 1–24 spectrin-like repeats, four hinges (H1–H4), a cysteine-rich domain, and a C-terminal domain (CTD). The nNOS-binding domain is shown by the red numbers (repeats 16 and 17).

Dystrophin structure-function studies have suggested that at least four SRs from the central rod domain are required to connect the crucial N-terminal and cysteine-rich domains. There are a variety of strategies to build such miniature dystrophins. Although it has been demonstrated that a variety of microdystrophins containing various SR combinations can alleviate the dystrophic pathophysiology, other SR arrangements have produced proteins with low or reduced functional capacity (Harper et al., 2002). For example, microdystrophin, ΔR4-R23/ΔCT (also known as mDysH2), one of the most used designs, reduces muscle necrosis and enhances muscle strength, but it has also been reported to cause ringbinden in some myofibers after myotendinous junction injury (Harper et al., 2002; Banks et al., 2008). Ringbinden was caused by a polyproline tract in hinge 2, which was corrected by replacing hinge 2 with hinge 3 (Banks et al., 2010). It is worth mentioning that Sarepta is testing this first-generation ΔR4-R23/ΔCT microdystrophin derived under the striated muscle-specific MHCK7 regulatory cassette in a human clinical trial (ClinicalTrials.gov: NCT03375164) (Figure 1) (Table 2) (Harper et al., 2002; Salva et al., 2007). (For more details, see *Clinical trials* section.)

**TABLE 2:** Clinical trials using adeno-associated virus- (AAV-) mediated microdystrophin transfer for DMD therapy.

Company	Solid biosciences	Sarepta therapeutics	Pfizer	Nationwide Children’s hospital
Description	Genetic: SGT-001	Genetic: rAAVrh74.MHCK7 microdystrophin	Genetic: PF-06939926	Administration of rAAVrh74.MCK.GAL GT2 to DMD patients
Clinical trial number	NCT03368742	NCT03375164	NCT03362502	NCT03333590
Trial title	Microdystrophin gene transfer study in adolescents and children with DMD (IGNITE DMD)	A randomized, double-blind, placebo-controlled study of SRP-90001	A study to evaluate the safety and tolerability of PF-06939926 gene therapy in DMD	Gene transfer clinical trial to deliver rAAVrh74.MCK.GALGT2 for DMD
Delivery route	Intravenous injection	Intravenous injection into peripheral arm vein	Intravenous injection	Intravascular
AAV serotype	AAV9	AAVrh74	AAV9	AAVrh74
Current stage	Active, but no longer recruiting participants	Active, but no longer recruiting participants	Active, but no longer recruiting participants	Active, not recruiting
Primary outcome	Safety	Safety	Safety and tolerability	Safety and tolerability

Several other mini-/microdystrophin designs with clinical potential have been generated and tested in animal models (Banks et al., 2007; 2010; Jørgensen et al., 2009; Lai et al., 2009; Koo et al., 2011; McCourt et al., 2015; Hakim et al., 2017; Le Guiner et al., 2017; Duan, 2018b; Nelson et al., 2018). Among these, Δ3990 (mini-dystrophin), ΔR4–23/ΔCT (microdystrophin with deletion of repeats 4–23 and deletion of the C-terminal domain), and R16/17-containing microdystrophin (μDys-5R) are particularly noteworthy given the high safety profile and potency of these constructs in the murine and canine models (Figure 1) (Wang et al., 2000; Harper et al., 2002; Hakim et al., 2017). Strategic design of these microgenes to increase therapeutic efficacy includes codon optimization—a modification that switches a particular DNA sequence by changing its codons to match the most prevalent tRNAs, resulting in a more efficient translation (Foster et al., 2008) and the inclusion of nNOS-binding domain (repeats 16 and 17) (Lai et al., 2009).

Although all microdystrophins currently in clinical trials include hinges H1 and H4 (Figure 1), Wasala et al. (2022) recently studied the importance of including hinge 1/4 in microdystrophin gene therapy. They reported that hinge 1 is essential for the correct sarcolemmal localization of microdystrophin, restores the DGC, and enhances specific muscle force significantly compared to hinge 4. However, the construct including H1 and H4 outperformed the H1-only or H4-only microdystrophins. The Chamberlain group has been a leader in the field of testing microdystrophins with different structural configurations, including modification with central rod and/or various hinge domains (Harper et al., 2002; Wang et al., 2012). Here, vectors were evaluated in mdx^4cv^ mice and delivered using recombinant AAV6 vector. When compared to first-generation microdystrophins, two of the new microdystrophins were found to improve force generation while also localizing neuronal nitric oxide synthase to the sarcolemma, namely, μDys5 (ΔR2-R15/ΔR18-R22/ΔCT) and μDys7 (ΔH2-R15 + H3/ΔR18-R23/ΔCT) (Ramos et al., 2019).

It is worth noting that studies are needed to fully investigate the stability, function, and proper localization of the several different micro/mini-dystrophin constructs, as it is not entirely clear if the proteins will localize correctly within the cytoskeletal network.

Additional research is required to assess how functional these microdystrophins are in humans, including their therapeutic effectiveness. A functional improvement in DMD patients may also be more challenging to predict, given that the DMD animal models (where preclinical studies are tested) frequently exhibit a milder skeletal and cardiac muscle phenotype than humans. Additionally, because microdystrophins are shorter than the typical length of truncated dystrophins in BMD patients, microdystrophin therapy in DMD patients may not fully achieve the goal of attaining a BMD-like phenotype (Mercuri and Muntoni, 2013). Accordingly, the loss of functionality owing to the deletion of specific dystrophin protein subdomains continues to be problematic in terms of treatment effectiveness. It will also be crucial to achieve significant therapeutic efficacy in the heart since treating skeletal muscle without also treating the associated cardiomyopathy could paradoxically add more strain on the myocardium, due to increased physical activity, and hasten the development of heart disease (Townsend et al., 2008; 2009). This may be a general concern with treatments that more specifically target skeletal muscles without also addressing heart tissue.

### 4.2 Exon-skipping therapy

**FIGURE 2:**
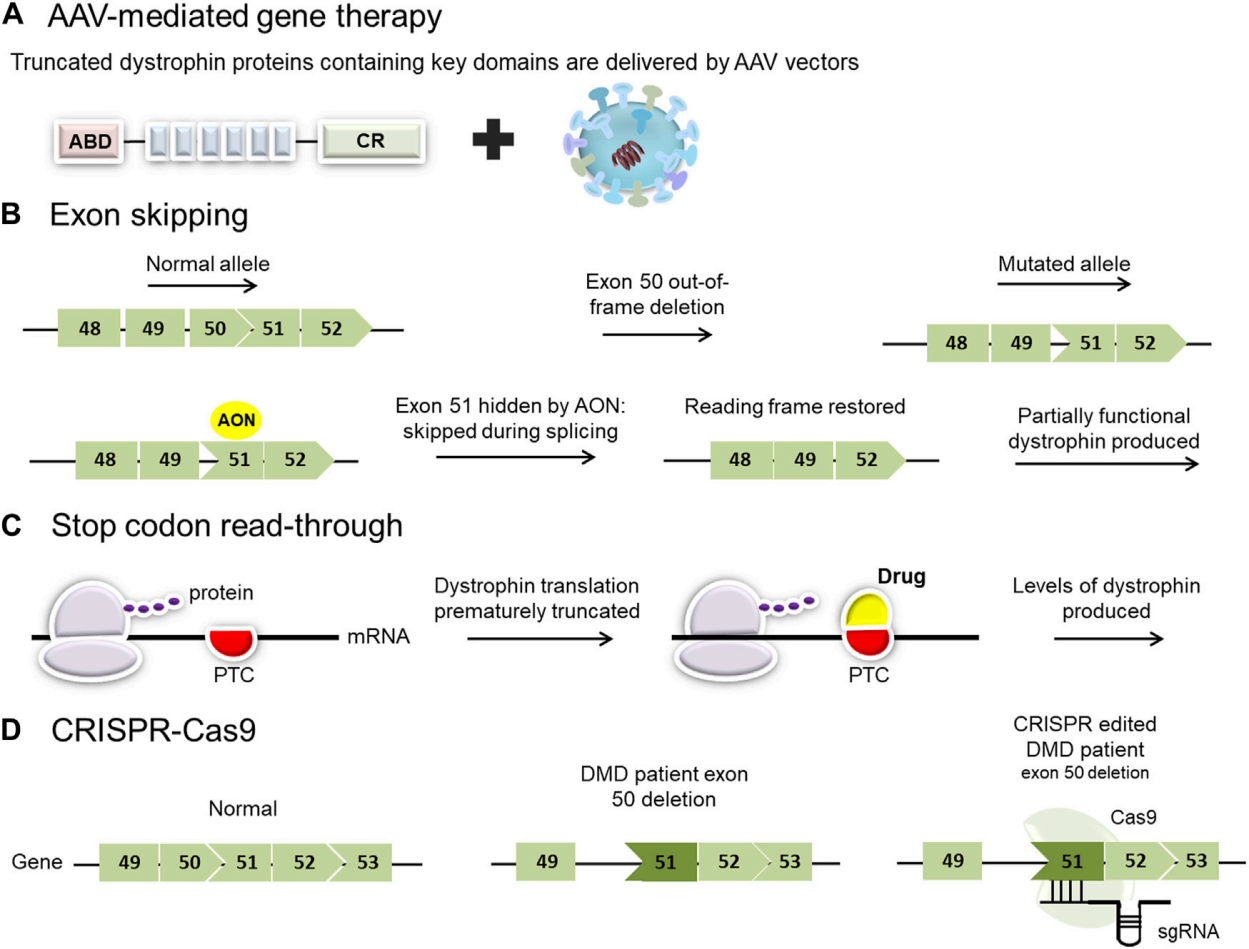
Overview of current and proposed experimental therapies for Duchenne muscular dystrophy (DMD). **(A)** AAV-mediated gene therapy employs viral vectors to deliver micro- or mini-dystrophin genes. Clinical trials using different adeno-associated virus (AAV) serotypes have shown promise for the treatment of patients with DMD. **(B)** Exon-skipping strategies seek to mask exons adjacent to others that have been deleted. This results in the restoration of the reading frame and permits the translation of a slightly smaller dystrophin product. **(C)** Stop codon read-through is a small molecule therapy aimed at reducing ribosomal sensitivity to mRNA stop codons, thus promoting ongoing dystrophin translation in those patients with nonsense mutations. (PTC: premature termination codon). **(D)** Genome editing, employing a CRISPR/Cas9 platform, has the potential to target specific pathogenic variants in the DMD gene but carries a risk of off-target effects.

Exon skipping has been proposed and studied as a possible treatment for DMD for more than two decades. The goal of exon skipping therapy is to restore the disrupted open reading frame (ORF) of the dystrophin gene transcripts in DMD patients, allowing them to make a Becker’s muscular dystrophy-like protein (Figure 2) (Niks and Aartsma-Rus, 2017). It is based on manipulating the pre-mRNA splicing of dystrophin transcripts to bypass a defective exon from the splicing machinery, restoring the reading frame and producing a partially functional, internally deleted dystrophin (Figure 2) (Schneider and Aartsma-Rus, 2021). The resulting dystrophin proteins are usually larger than the majority of mini- and microdystrophin gene therapy constructs currently under investigation.

Exon-skipping entails three experimental approaches: CRISPR DNA editing system, U7 snRNP-mediated splice blocking, and antisense oligonucleotides (AONs) (Takeda et al., 2021). CRISPR DNA editing techniques work at the DNA level to change myofiber genomic DNA in converting a DMD out-of-frame deletion into a BMD-like in-frame deletion. CRISPR relies on viral vectors to deliver the DNA editing technology (for more information on CRISPR-mediated exon skipping, see *CRISPR-Cas9 mediated gene editing strategies*). U7 snRNP-mediated inhibition of splicing is another approach for exon-skipping where modified U7 snRNP genes are delivered via AAV vectors (Vulin et al., 2004; Gadgil and Raczyńska, 2021). In this instance, an antisense sequence that targets the exon takes the place of the typical antisense portion that hybridizes to histone RNA. This does not target mRNA but pre-mRNA (similar to the mechanism of AON) (Gushchina et al., 2021). The third approach to accomplishing exon skipping is using oligonucleotide drugs that bind to the pre-mRNA (prior to splicing) to modulate RNA splicing. These oligonucleotide techniques have used a variety of drug chemistry with variable success, and this approach will be the focus of the following section.

#### 4.2.1 AONs-mediated exon-skipping therapy

AONs are small 20–30 nucleotides of modified DNA or RNA homologs that specifically bind to their target exon prior to pre-mRNA splicing in a Watson-Crick-like manner, hiding the target exon from the splicing machinery so that it is spliced out with its flanking introns blocking its incorporation to the mature mRNA (Schneider and Aartsma-Rus, 2021). Antisense-mediated exon skipping is a method that targets a specific mutation (Bladen et al., 2015). For the reading frame to be restored, different exons have to be skipped depending on the location and size of the mutations (Aartsma-Rus et al., 2009; Bladen et al., 2015). Accordingly, different dystrophin proteins will be generated after skipping different exons for different mutations. Hence, it is crucial to have a precise genetic diagnosis of the disease to be able to design effective AONs (Arechavala-Gomeza et al., 2007).

For AON therapy, the majority of DMD patients would have one or more exons deleted. Overall, in DMD patients, 70% of the reading frame can be restored by single exon skipping, whereas another 8% would benefit from double exon skipping to restore the reading frame (Bladen et al., 2015). DMD deletions frequently cluster in hotspots between exons 45 and 55; therefore, skipping target exons in this area could be of benefit to a large group of patients. Certain exons have been reported to have the highest applicability for skipping. Namely, exon 51 could be applied for (13%–14%) of patients, exon 45 and exon 53 both could be skipped for another 8%–10% of patients, and exon 44 could be skipped for an additional 6% of the patients (Mann et al., 2001; Van Deutekom et al., 2001).

Different chemical modifications have been tested to give the AONs drug-like characteristics in trying to enhance their bioavailability, improving their resistance to exonucleases and endonucleases, and increasing their affinity to the target RNA transcripts (Saleh et al., 2012). 2ʹ-O-methyl phosphorothioate (2OMePS) AONs and phosphorodiamidate morpholino oligomers (PMOs) are used among other chemistries for DMD exon skipping clinical development approaches (Verma, 2018; Schneider and Aartsma-Rus, 2021).

A major challenge for the success of AON approaches centers on achieving adequate delivery to the myofiber nuclei so that the drug can hit its pre-mRNA target in the nucleus (prior to splicing) and prevent the splicing of the targeted exon (Takeda et al., 2021). Three different techniques have been used for the delivery of the oligonucleotides to the myofiber, namely, cell transfections, *in vivo* intramuscular injections, and *in vivo* systemic delivery (intravenous, intraperitoneal, or subcutaneous). Depending on the delivery technique, various oligonucleotide chemistries exhibit different degrees of myofiber delivery efficacy and exon-skipping efficiency (Heemskerk et al., 2009; Hoffman et al., 2011; Takeda et al., 2021).

The use of nanoparticles as a delivery vehicle has been investigated for enhancing AON uptake and reducing possible immune reactions. Polymethyl methacrylate (PMMA) nanoparticles include two subgroups: T1 and ZM2 have been tested for AON therapy in mdx mice (Schneider and Aartsma-Rus, 2021). T1 nanoparticles were shown to have slow biodegradability; however, they formed aggregates that precluded their use via intravenous administration and raised concerns about possible adverse effects (Rimessi et al., 2009). Regarding ZM2-encapsulated AONs, intraperitoneal delivery was accompanied by higher dystrophin restoration levels in skeletal muscle and hearts as compared to naked 2OMePs AONs (Rimessi et al., 2009; Ferlini et al., 2010). Moreover, the effect lasted for 90 days after the course of treatment (7 weeks) (Bassi et al., 2012). Still, more studies are needed to better understand the pharmacodynamics, pharmacokinetics, and any related side effects of this type of delivery.

2OMePS molecules possess a negative charge. They can effectively transfect cells *in vitro* and can be absorbed via intramuscular injection. Unfortunately, their use in clinical trials has been stopped, as no detectable dystrophin production in patient muscle was observed (Mann et al., 2001; Lu et al., 2003). Morpholinos (PMO) are uncharged compounds that are challenging to introduce into cells *in vitro*. They show little or no systemic distribution to normal myofibers but exhibit high levels of systemic delivery and efficient exon skipping in dystrophic muscle (Heemskerk et al., 2009; Hoffman et al., 2011; Novak et al., 2017). The effective delivery of PMOs was limited to the inflammatory foci and active muscle-regenerating regions of the dystrophic muscle tissue, where the macrophages seem to play a role in their delivery (Novak et al., 2017). Morpholinos have shown an acceptable safety profile at very large systemic doses in mouse, dog, and human studies (Yokota et al., 2009; Wu et al., 2010; Clemens et al., 2020; Komaki et al., 2020). Nevertheless, as the underlying mutations that cause DMD disease remain and due to transcript and protein turnover, repeated drug injection is required. Overall, the highest levels of dystrophin restoration have been achieved via morpholino chemistry using systemic delivery (Schneider and Aartsma-Rus, 2021).

Several strategies have been proposed to increase the effectiveness of AONs and their uptake by skeletal muscles and in the heart for multi-exons skipping or enhancing their delivery using muscle-homing peptide conjugation (e.g., arginine-rich peptide) or via nanoparticles. Multi-exon skipping approaches, which permit the skipping of several adjacent exons at once, are under investigation in order to broaden the range of mutations eligible for treatment (Goyenvalle et al., 2012). Cocktails of antisense oligonucleotides have been developed to target two or more neighboring exons within a particular area to induce multi-exon skipping, such as the major mutational hotspots (exons 45–55) (Taglia et al., 2015). Another favorable approach is conjugating AONs to arginine-rich cell-penetrating peptides (CPPs) to enhance cellular uptake. The conjugated PMO-based AONs are termed peptide phosphorodiamidate morpholino oligomers (PPMO). In mdx mice and canine X-linked muscular dystrophy model of DMD, PPMOs were shown to be safe and permitted a longer duration of effective exon skipping in the tissues evaluated, including the heart and diaphragm (Jearawiriyapaisarn et al., 2008; Wu et al., 2008; Echigoya et al., 2017).

Despite the attractiveness and applicability of single or multiple exon(s) skipping techniques and the fact that four AONs have already been approved for clinical trials, it is becoming clear that their delivery efficiency is not satisfactory (Sheikh and Yokota, 2021). This could be concluded from the low levels of dystrophin restoration in skeletal muscle biopsies and the lower levels observed in heart muscle (Heemskerk et al., 2009; Wu et al., 2009; Heemskerk et al., 2010; Jearawiriyapaisarn et al., 2010; Wu et al., 2010; Malerba et al., 2011; Yin et al., 2011). Therefore, ultimate clinical effectiveness will necessitate repeated injections. For exon skipping to reach clinical milestones, drug-induced dystrophin needs to be produced at higher levels in both skeletal and heart muscles. Hence, it is crucial to understand the underlying pathogenesis to maximize the treatment benefit. Epigenetic modulators that enhance or abolish the inhibitory effect of gene expression need to be considered with this treatment to retain and stabilize dystrophin transcripts and protein at multiple levels.

Taken together, the exon-skipping technique has advanced from *in vitro* proof of concept research of the clinical trial stage to FDA approval (Eser and Topaloğlu, 2022). Only single exon skipping has been approved for clinical trials (for more details, see *Clinical trials* section). The multiple exon-skipping approach is still facing hurdles (e.g., higher drug doses required, differential uptake and efficiency of the used ASOs) (Mitrpant et al., 2009; Yokota et al., 2009; Aartsma-Rus et al., 2014).

### 4.3 Stop codon read-through

Around 10%–15% of patients with DMD have a nonsense mutation that induces a premature termination codon (PTC) in the mRNA, causing the ribosome to terminate translation and failing to synthesize the remainder of the protein (Flanigan, 2014; Bladen et al., 2015). The principle of stop codon read-through is to induce the ribosome to continue translating the mRNA through the premature stop codon and continue through the rest of the transcript. The drug works by binding to the ribosome and its partners, disrupting its ability to recognize the premature stop codon, leading to the transformation of the PTC into a correct codon, thereby resulting in the continuation of the translation (Figure 2) (Bordeira-Carriço et al., 2012). This mechanism ideally needs to be specific to premature stop codons, without read-through of bona fide stop codons, to efficiently enable partial restoration of dystrophin expression in muscle (Welch et al., 2007). A number of drugs can induce stop codon read-through, and their efficacies rely on the nature of the nonsense mutation and the surrounding nucleotide sequences. These drugs can be divided into two main groups: aminoglycoside antibiotics (e.g., gentamycin; amikacin, tobramycin, and paromomycin) or chemical compounds without known analogs (e.g., ataluren or negamycin) (Bidou et al., 2012). Although gentamycin demonstrated efficacy in inducing stop codon read-through for numerous pathologies, including DMD (Barton-Davis et al., 1999; Wagner et al., 2001; Politano et al., 2003; Malik et al., 2010), toxicity remains a major concern limiting gentamycin from clinical considerations (Prayle and Smyth, 2010; Beringer and Winter 2011). In the past few years, several studies have aimed to select molecules capable of promoting mRNA translation despite the presence of a PTC. Ataluren (Translarna TM, previously known as PTC124) is a small oral molecule developed by PTC Therapeutics as a potentially safer alternative to induce stop codon read-through (Welch et al., 2007).

### 4.4 CRISPR-Cas9 mediated gene editing strategies

Over the past decade, gene editing has moved to the forefront with the identification of mechanisms of DNA repair (Stephenson and Flanigan, 2021). The CRISPR-Cas system has made it possible for research labs all around the world to successfully incorporate gene editing into their work (Jinek et al., 2012; Mali et al., 2013; Perez-Pinera et al., 2013). The two main components of the CRISPR/Cas9 system are the CRISPR-associated (Cas) endonuclease and a single guide RNA (sgRNA), which directs Cas9 to a particular ∼20-nucleotide region in the genome that contains the complementary sequence (Figure 2).

Numerous engineered Cas endonucleases have been generated using naturally occurring Cas enzymes as guides. Targeted modifications have improved enzyme fidelity, reduced off-target mutations, increased editing efficiency, and used a more dispensable PAM sequence (Mali et al., 2013; Makarova et al., 2020). Engineered Cas proteins have extended to include nickase Cas9 (nCas9) (Ran et al., 2013) and deactivated Cas9 (dCas9) (Qi et al., 2013) that have been engineered to retain their programmable DNA-binding ability while decreasing or abolishing the Cas9 endonuclease activity, respectively. This has increased the safety of the CRISPR/Cas9 system owing to the absence of any DSBs (Dominguez et al., 2016). These may be combined with other elements, such as precise base editors (Komor et al., 2016; Yuan et al., 2018) or transcriptional activators (Qi et al., 2013; Wojtal et al., 2016), for alternative therapeutic strategies (Chen et al., 2022). Thus, the toolkit of CRISPR gene-editing systems has advanced, providing a growing number of options for CRISPR-based therapies.

The potential treatment of DMD using CRISPR/Cas9-mediated therapeutic techniques and the difficulties of CRISPR/Cas9-mediated therapeutic genome editing will be the main topics of this section.

#### 4.4.1 CRISPR technology as a gene-editing therapeutic for DMD

A single infusion of CRISPR genome-editing components can theoretically treat DMD by correcting genetic mutations at the genome level utilizing CRISPR/Cas programmable nucleases (Olson, 2021; Chen et al., 2022), a concept that represents a promising therapeutic strategy for the long-term correction of genetic illnesses (Knott and Doudna, 2018). Several of the disease characteristics of DMD make gene editing an exciting treatment modality. First, owing to the relative dispensability of certain dystrophin domains, it is possible to remove mutant exons from the rod domain of the gene and restore the ORF yielding partially functional truncated dystrophins, as discussed previously. Second, a genetic repair of even a small percentage of muscle nuclei could enable the generation of dystrophin and its dissemination throughout the myofibers as skeletal fibers are multinucleated. Moreover, because the dystrophin gene is located on the X chromosome, affected boys only have one mutant allele that needs to be repaired. Therefore, unintentionally altering a wild-type copy of the gene is obviated (Long et al., 2014).

Both *in vitro* and *in vivo* studies using CRISPR-mediated gene editing for DMD have been conducted. Therapeutic effects have been reported in human cells and mice, rats, dogs, and piglets with various DMD mutations (Ousterout et al., 2015; Young et al., 2016; Amoasii et al., 2018; Duchene et al., 2018; Min et al., 2019a; Moretti et al., 2020; Pickar-Oliver et al., 2021; Szabo et al., 2021; Zhang et al., 2021). Attaining greater insights using sequence-humanized animal models will be required prior to any clinical translation. This would include providing crucial information such as the required dose, delivery vehicle, route of administration, and the percentage levels required for functional dystrophin recovery (Aartsma-Rus and van Putten, 2020; Olson, 2021). Off-target effects are also a concern and must be addressed prior to human trials.

#### 4.4.2 Exon deletion

Exon excision, exon skipping, exon reframing, and exon knock-in are the main approaches for the CRISPR-mediated therapeutic repair of DMD mutations. Additionally, newly engineered CRISPR system technologies like the base or prime editors have been used to enable more accurate gene editing.

The majority (∼60%) of DMD patients have a deletion of one or more exons. Deleting the out-of-frame exon to restore the ORF is a typical method for achieving single or multiple exon deletions. This can be accomplished by double-cut exon deletion to one exon or over the entire hotspot region. To cut out single or multiple mutant exons, two sgRNAs flanking these sites can be targeted with Cas9, and this will cause the ORF to be restored by splicing the neighboring in-frame exons together (Ousterout et al., 2015; Maggio et al., 2016a; Young et al., 2016). Importantly, because this approach is less mutation-specific and permits the ablation of many exons in a mutational hotspot, it is applicable to a higher percentage of DMD patients. However, this is at the expense of shorter dystrophins compared to other correction strategies. This method, though appealing, has a number of drawbacks, including the increased risk of off-target mutations and a sharp decline in editing efficiency simply because two directed DSBs must occur at the same time for the edit/correction to be successful. Added to this issue is the challenge of delivering the two sgRNAs simultaneously.

DMD mutational hot spot deletion has the potential to cure around 60% of DMD patients, based on BMD patients with more mild symptoms, at least in skeletal muscles (Aartsma-Rus et al., 2006; Doo et al., 2012; Ousterout et al., 2015). Ousterout et al. (2015) designed multiplexed sgRNAs to restore the dystrophin reading frame by targeting the mutational hotspot in ∆Ex48-50 DMD patient-derived myoblasts. Here, dystrophin expression was restored *in vitro* following gene editing. However, this correction was not as efficient as deleting exon 51 alone, and this raised concerns about the editing efficiency for deleting a larger DNA fragment. Other groups targeted this same region *in vitro* and demonstrated a successful restoration of dystrophin in multiple DMD cell lines (Maggio et al., 2016b; Young et al., 2016; Duchene et al., 2018). *In vivo* studies have also been conducted in the humanized DMD del45/mdx mouse model. Exons 45–55 were successfully deleted, and dystrophin expression was observed after gene correction (Young et al., 2017).

*In vitro* and *in vivo* studies using the double-cut gene-editing approach have been used to target single-exon deletions (Maggio et al., 2016b; Long et al., 2016; Tabebordbar et al., 2016; Zhang et al., 2017; Long et al., 2018; Matre et al., 2019; Moretti et al., 2020). Although removing a smaller region lessens some of the problems with the double-cut method, it also eliminates the multi-exon deletion advantages noted previously. Postnatal single exon deletion editing has been studied in the mdx mouse model. Here, dystrophin expression was partially restored in cardiac and skeletal muscle and was shown to last up to 1 year in some studies after a single intravenous injection of an adeno-associated virus that encodes the CRISPR cassette (Nelson et al., 2016; Nelson et al., 2019).

Exon duplication mutations, which affect 5% of DMD patients, can also be corrected using exon deletion techniques. One duplicate exon can be targeted with a single sgRNA designed against the intron region next to the duplicated exon. In the presence of Cas9, sgRNA will produce two cuts and eliminate one of the duplicate exons. Thus, in principle, the dystrophin gene ORF can be restored, resulting in the production of full-length dystrophin protein identical to normal dystrophin (Long et al., 2018). However, exon duplication excision is presently challenging to investigate *in vivo* owing to the lack of animal models with this mutation (Bladen et al., 2015). CRISPR/Cas9 technology was recently used to develop a mouse model with a muti-exon tandem duplication of exon 50. This duplication mutation was then corrected using the sgRNA CRISPR/Cas9 technique. This method removed the duplication mutation, restored the expression of full-length dystrophin, and improved muscle functionality *in vivo* (Maino et al., 2021). Thus, this approach has the potential to treat duplication mutations in DMD.

#### 4.4.3 Exon skipping by gene editing

In comparison to AON-based exon skipping therapy that modulates dystrophin mRNA, CRISPR gene editing corrects the underlying mutation in the genome. Thus, it is regarded as a permanent genomic correction. The conservative CRISPR-based exon skipping approach using only one sgRNA, instead of two sgRNAs flanking a mutant exon, to abolish either the splice acceptor site or splice donor site of the out-of-frame exon is the commonly used method for exon skipping (Amoasii et al., 2018). The sequence encoding the exon splice acceptor or donor site is then deleted by NHEJ induced via CRISPR system-triggered DSB, leading to out-of-frame exon skipping and splicing to the next accessible exon. Compared to utilizing two sgRNAs to flank the exon for removal, a single-sgRNA method for exon skipping increases editing efficiency (Long et al., 2018).

Several *in vitro* and *in vivo* studies have been conducted to evaluate this approach. *In vitro*, single-cut exon skipping has been used to skip exons 43, 45, 51, and 53 by NHEJ-induced disruption of splice acceptor sites, and successful skipping was demonstrated in iPSCs-derived skeletal muscle myoblasts isolated from DMD patients. Dystrophin restoration and functional improvement were observed in the gene-edited cell lines (Maggio et al., 2016b; Long et al., 2018; Min et al., 2019b). This approach has also been tested *in vivo* in both mouse (Amoasii et al., 2017; Min et al., 2019b; Wei et al., 2020) and canine models (Amoasii et al., 2018). Targeting the splice acceptor site of exon 51 results in skipping exon 51 and restoration of the dystrophin ORF. Dystrophin expression restoration and improved muscle function were demonstrated in both the DMD mouse and canine models that lack exon 50 (Amoasii et al., 2017; Amoasii et al., 2018). Exon 45 skipping via CRISPR/Cas9 has also been demonstrated to restore the reading frame in the ∆Ex44 mouse model, allowing exon 43 to splice to exon 46, thus restoring the dystrophin reading frame (Nelson et al., 2017; Min et al., 2019b).

#### 4.4.4 Exon reframing by gene editing

Another strategy to restore the dystrophin ORF involves Cas9 induction of a single NHEJ (Li et al., 2015; Iyombe-Engembe et al., 2016; Amoasii et al., 2017; Long et al., 2018). This form of gene editing is designed to “reframe” the ORF of the dystrophin transcript by introducing small insertions and deletions (INDELS) via NHEJ of double-stranded DNA breaks generated by CRISPR-Cas9. When using a sgRNA to induce NHEJ in an out-of-frame exon, the generated indels result in a targeted frameshift to restore the dystrophin gene back in frame (Min et al., 2018). This method is often referred to as single-cut myoediting because only one sgRNA is used to direct Cas9 editing, requiring only a single cut to restore the dystrophin ORF in targeted muscle cells. A number of groups have shown efficient restoration of the dystrophin ORF through exon reframing in human iPSC-derived cardiac myocytes, mouse models, and large animal models of DMD (Ousterout et al., 2015; Amoasii et al., 2017; 2018; Bengtsson et al., 2017; Kyrychenko et al., 2017; Zhang et al., 2017; Long et al., 2018; Yuan et al., 2018). Exon reframing produces small indels during the repair and serves as an efficient strategy to preserve a large portion of the dystrophin genomic sequence while bypassing the DMD mutation. This strategy offers the possibility of permanently correcting specific DMD mutations.

#### 4.4.5 Exon knock-in

In contrast to NHEJ-mediated exon deletion, skipping or reframing HDR-mediated exon knock-in can restore the full-length dystrophin gene expression. This approach incorporates a DNA donor template with the appropriate sequence as a part of the editing components. HDR-mediated genome editing has been demonstrated in small and large animal models of DMD (Long et al., 2014; Bengtsson et al., 2017; Zhang et al., 2017; Zhu et al., 2017; Mata López et al., 2020). An example of this strategy is the correction of exon 23 in the germline of mdx mice via SpCas9 with a 180-nt single-stranded DNA oligonucleotide template. Correction rates ranged from 2% to 100% correction of the *Dmd* gene in the resultant mosaic mice (Long et al., 2014). HDR is not active in quiescent or G1-arrested cells, rendering it unsuitable in mature myofibers and cardiac myocytes. Additionally, the approach cannot be used to correct DMD deletion mutations because of the length restriction on the donor DNA template (Zhang et al., 2021).

Homology-independent targeted integration (HITI) is a gene editing method with a relatively high efficiency in postmitotic cells. HITI can accurately knock in a missing exon(s) at a specific locus using NHEJ, which bypasses the requirement of HDR. HITI has been developed to overcome the HDR-related challenges noted previously (Suzuki et al., 2016). Delivering a donor plasmid with two Cas9 cleavage sites flanking the desired donor sequence is a key component of HITI. Following the Cas9 cleavage of the targeted genomic DNA and the donor plasmid, the NHEJ repair pathway will then incorporate the donor sequence. Although this exon knock-in strategy to restore full-length dystrophin protein is promising, it has not yet been extensively evaluated in the setting of DMD. Pickar-Oliver et al. examined the HITI-mediated approach to insert the missing human exon 52 or a superexon encoding the human dystrophin cDNA sequence downstream of exon 51 into its corresponding position within the dystrophin gene in a humanized mouse model of DMD. The DMD model used contained an out-of-frame deletion of exon 52, and full-length dystrophin restoration in skeletal and cardiac muscles was demonstrated (Pickar-Oliver et al., 2021). This technique enables the entire restoration of full-length dystrophin, even though the insertion efficiencies were low, and would be applicable to about 20% of DMD patients.

#### 4.4.6 Base editing

Roughly 25%–30% of patients with DMD have point mutations (Aartsma-Rus et al., 2006; Bladen et al., 2015). Base editing is a recently developed approach to expand the toolbox of gene editing strategies to treat DMD (Chemello et al., 2020). In base editing, there are two major categories of DNA base editors: cytosine base editors (CBEs), which convert the C:G base pair into a T:A base pair (Komor et al., 2016; Nishida et al., 2016; Gaudelli et al., 2017) and adenine base editors (ABEs), which convert A:T base pairs to G:C base pairs (Gaudelli et al., 2017). In this strategy, Cas9 nickase (nCas9) or deactivated Cas9 (dCas9) is fused to a cytidine deaminase or an engineered adenine deaminase protein, allowing precise single-base pair conversations without double-stranded breaks (Gaudelli et al., 2017; Huang et al., 2021). These RNA-guided nucleotide-specific base editors do not rely on the NHEJ repair pathway, and as a consequence, small indels through error-prone NHEJ at the target site are not produced. Furthermore, a donor DNA template for HDR is not required (Min et al., 2018). Recently, a CRISPR/Cas9 adenine base editor (ABE7.10) was used to substitute a single adenine with guanine in a DMD mouse model containing a nonsense mutation on exon 20 (Ryu et al., 2018). This strategy has also been used to induce exon skipping by mutating target DNA bases of splice motifs (Gapinske et al., 2018). In this context, a CBE base editor (hAID P182X) was implemented in various canonical intronic motifs to modulate the splicing of different genes (Yuan et al., 2018).

#### 4.4.7 Prime editing

More recently, a new strategy, termed prime editing, has been developed, which has been added to the CRISPR techniques to treat DMD point mutations (Anzalone et al., 2019). Prime editing takes advantage of a catalytically inactive nCas9. As a result, no DSBs are produced. The nCas9 is coupled to an engineered reverse transcriptase and delivered with an extended sgRNA termed the “prime editing” guide RNA (pegRNA). Reverse transcriptase uses the pegRNA as a template to add a DNA alteration at the target location (Anzalone et al., 2020). This approach enables single-base transitions or transversions, as well as site-specific genomic insertions and deletions, without introducing DSBs or the need for an exogenous donor DNA as a template for HDR repair. With the aid of endogenous DNA repair pathways, this newly generated DNA flap is then incorporated into the genome. While size restrictions still pose a problem for *in vivo* distribution, prime editing provides the ability to correct a number of DMD-causing mutations. In a proof-of-concept study by Chemello et al. (2021), prime editing was tested in the context of DMD. The approach was shown to be capable of reframing the dystrophin ORF in DMD (∆Ex51) cardiac myocytes derived from human iPSCs (Chemello et al., 2021). Subsequently, the Tremblay group reported the correction of point mutations in exon 6 in human DMD myoblasts using the prime editing technique (Happi Mbakam et al., 2022b).

#### 4.4.8 Delivery of CRISPR *in vivo*

An effective delivery mechanism is necessary to accomplish efficient *in vivo* postnatal genome editing. Cas9 and sgRNA, the two components of the CRISPR system, can be administered to the target organs via a variety of forms. DNA/DNA, mRNA/sgRNA, and protein/sgRNA are the possible nucleotide forms for Cas9 and sgRNA, respectively. The CRISPR delivery strategies frequently employed in DMD include viral and nonviral methods (Min et al., 2019a).

##### 4.4.8.1 Viral delivery

For the delivery of CRISPR/Cas9 components, lentivirus, adenovirus, and adeno-associated virus (AAV) have been employed (Min et al., 2019a), and clinical trials using AAV for gene replacement therapy have been approved by the FDA (Mendell et al., 2017). AAV serotypes 1, 6, 8, 9, rh10, and rh74 have tropism for skeletal muscle and heart. These serotypes have been successfully used in numerous preclinical investigations to deliver CRISPR gene editing components for postnatal genome editing (Lau and Suh, 2017; Wang et al., 2017).

The SpCas9 ORF is about 4.2 kb in length, which is close to the AAV cargo limit. Thus, a second AAV vector that carries the donor template or sgRNA is required. Hepatocytes and muscle cells have been successfully edited *in vivo* using a dual-AAV approach (Yang et al., 2016). *Staphylococcus aureus* (Sa) Cas9, a smaller Cas9 protein encoded by a 3.2-kb cDNA, has been employed for gene editing in mdx mice to avoid the requirement for a dual-vector system (Nelson et al., 2016; Tabebordbar et al., 2016; Bengtsson et al., 2017). For more flexibility with promoter usage in DMD gene therapy, new AAV serotypes have been engineered, such as the MyoAAV 2A serotype, which appears more efficient than AAV9 in muscle fibers. This serotype can be employed with genome-specific modifiers, including base or prime editors, to maximize therapeutic potential (Mendell et al., 2020; Tabebordbar et al., 2021; Happi Mbakam et al., 2022a).

##### 4.4.8.2 Nonviral delivery

Cas9 and sgRNAs can be delivered *in vivo* through nonviral delivery mechanisms in a variety of forms, including DNA, mRNA, and ribonucleoproteins (RNP). Electroporation has been used to deliver negatively charged DNA or mRNA into muscle cells (Xu et al., 2016). Direct delivery of Cas9 and sgRNA constructs into the skeletal muscle of mdx mice using this technique led to the restoration of dystrophin expression (Xu et al., 2016). Another delivery method for CRISPR components, such as RNP or mRNA/sgRNA, is via lipid-mediated nanoparticles (Zuris et al., 2015; Miller et al., 2017). The cationic lipid nanoparticles can enclose the complex, which can then be transported into cells via endocytosis and pinocytosis. This method is relatively inexpensive compared to other approaches (Kim et al., 2014). Gold nanoparticles coupled to DNA and complexed with cationic endosomal disruptive polymers have also been reported to successfully deliver CRISPR RNP to mdx mice (Lee et al., 2017). Even though it seems to be an appealing strategy for delivering the RNP complex, a major challenge is the systemic delivery requirement to reach the heart, diaphragm, and skeletal muscles through the body. This, together with issues of efficiency, needs to be addressed before effective clinical applications can be investigated.

#### 4.4.9 Gene editing: clinical considerations

Theoretically, gene editing has the potential to cure the disease with a single treatment, “one and done,” by correcting the genetic defect causing the condition. Gene editing can enable the expression of normal or nearly normal dystrophin to maximize functional outcomes. The resultant dystrophin, post-gene editing, is anticipated to be larger than microdystrophins which are undergoing clinical trials. Physiological temporal-spatial expression is expected to be normal because the expression of dystrophin after gene editing will be driven under the control of the endogenous dystrophin gene locus. This is compared to therapeutic microdystrophin gene therapy, where expression is dependent on the cis-regulatory sequences designed and incorporated in the construct within the AAV vector (Min et al., 2019b).

#### 4.4.10 Gene editing: future challenges

Despite recent advances, the gene-editing experimental approach is still in the early stages of development for DMD therapy, with several concerns that should be addressed. Notably, off-target mutation risk *in vivo* is a significant concern. Muscle-specific promoters have been used to drive the CRISPR cassette expression to reduce possible off-target effects in non-muscle tissues (Young et al., 2016; Miller et al., 2017). Other issues to be addressed are the delivery efficiency to all the affected skeletal muscles and the heart, in addition to issues related to the maintenance of long-term dystrophin expression. Cardiac myocytes are long-lived cells with minimal regeneration capacity; thus, upon adequate delivery of the editing cassette and precise correction, myocytes are anticipated to exhibit long-term dystrophin expression. However, skeletal muscle fibers have a remarkable regeneration capacity (Ma et al., 2022). Skeletal muscle cells are generated via satellite cell recruitment, the muscle stem cells that are not efficiently transduced by AAV (Arnett et al., 2014; Min et al., 2019b). Thus, the skeletal edited muscle cell proportion will be diluted with every regeneration cycle, ultimately reducing dystrophin expression in the fibers.

Furthermore, there are a number of immunological issues to take into account, such as immunogenicity toward Cas protein or the likelihood of immune reaction toward the replaced dystrophin, a possibility that remains with all gene replacement therapies. More research is required to fully address these concerns (Min et al., 2019b). Thus, further studies focusing on the safety and efficiency of the gene editing CRISPR system are necessary prior to clinical translation (Erkut and Yokota, 2022). The CRISPR system has recently been introduced into clinical trials for other diseases, such as cancer; allergy; and cardiovascular, immunological, and neurological disorders (Sharma et al., 2021). Even though the CRISPR system faces several challenges, it has undoubtedly created new possibilities for treating monogenic diseases that will certainly pave the way for many applications in the upcoming years.

### 4.5 Cell transplantation therapy

DMD cell-based therapeutics aim to transplant cells with functional dystrophin into dystrophin-deficient muscles. This is one of the earliest genetic approaches attempting to treat DMD (Smythe et al., 2000; Skuk et al., 2004; Skuk et al., 2006). However, multiple factors must be considered for successful muscle recovery. Autogenic cell transplantation, relying on genetic engineering of the host’s induced pluripotent stem cells (iPSCs) to restore functional dystrophin expression, is associated with lower immunogenic risk than allogenic cell transplantation (obtained from a healthy donor) requiring immunosuppression (Maffioletti et al., 2014). Ideally, the transplanted cells should be competent to cross the vascular wall (blood–muscle barrier) upon systemic delivery to target different striated muscle entities (heart, diaphragm, and limb muscles) to limit the requirement for multiple injection sites into individual muscles. Moreover, to provide a long-term effect and reduce the risks of inducing an immune response from repeated deliveries, the transplanted cells must have a high muscle engraftment rate, incorporate into the host myocytes, and persist through self-renewal (Wallace et al., 2008).

Various cell types are candidates for transplantation and are being studied for DMD therapy. Satellite cells (myoblasts) are quiescent muscle stem cells that reside beneath the basal lamina (Mauro, 1961; Moss and Leblond, 1971). Upon muscle damage, myoblasts are activated to proliferate, self-renew, and repair. Other cell types, notably bone marrow-derived cells, pericytes, mesoangioblasts, and mesenchymal stem cells, are also under investigation because of their ability to access the muscle compartment and their myogenic potential. However, the limited capacity of these cells to be expanded *in vitro* constitutes a limitation for wide therapeutic usage. Nonetheless, based on the early successes in preclinical studies, a clinical trial is underway to transplant skeletal muscle-derived mesoangioblasts into the circulation of patients with DMD. Induced pluripotent stem cells (iPSCs), featuring an infinite self-renewal capacity, can be differentiated into myogenic lineages. However, iPSCs-derived myogenic progenitors could carry the risk of unlimited growth and teratoma development *in vivo* (Rong et al., 2012).

One of the most challenging hurdles for cell-based clinical applications centers on delivery. Although preclinical studies show promise (Rouger et al., 2011), most systemic studies report only limited engraftment of dystrophin restoration (Briggs and Morgan, 2013; Hogrel et al., 2013). Myoblasts lack extravasation ability, and continuous efforts are being made to improve their trans-endothelial migration ability (Choi et al., 2022). As for extravasation-competent stem cells, only a small fraction of transplanted stem cells appears to reach the muscle upon systemic delivery (Dellavalle et al., 2007). Additionally, although local high-density myoblast injections (intra-muscular injections) produce some dystrophin restoration around the needle track (Skuk et al., 2004; 2006), this approach is only practical for superficial small muscles.

On another note, cardiosphere-derived stem cells offer a transplantable cell type with therapeutic potential. Preliminary results from the randomized, phase I/II HOPE-Duchenne clinical trial (NCT02485938) revealed that the intra-coronary infusion of cardiosphere-derived cells was well tolerated and suggested a positive effect on upper limb and heart function for up to 12 months (Taylor et al., 2019). Furthermore, phase II of the Hope-2 clinical trial (NCT04428476) recently revealed that intravenous delivery of cardiosphere-derived cells is safe and has beneficial effects in slowing the deterioration of muscle function in patients with late-stage DMD (McDonald et al., 2022).

## 5 Small molecule therapy for DMD: copolymers

Despite the numerous trials exploring potential therapeutics, there remains a dearth of effective treatments available for DMD patients. In this context, it is worth considering additional strategic approaches that target the primary defect of DMD: severe muscle membrane fragility. As discussed previously, as the primary pathophysiological defect in DMD is the marked susceptibility to contraction-induced membrane stress, a unique therapeutic approach is the use of synthetic membrane stabilizers to prevent muscle damage by directly stabilizing the dystrophin-deficient muscle membrane (Houang et al., 2015; 2017).

**FIGURE 3:**
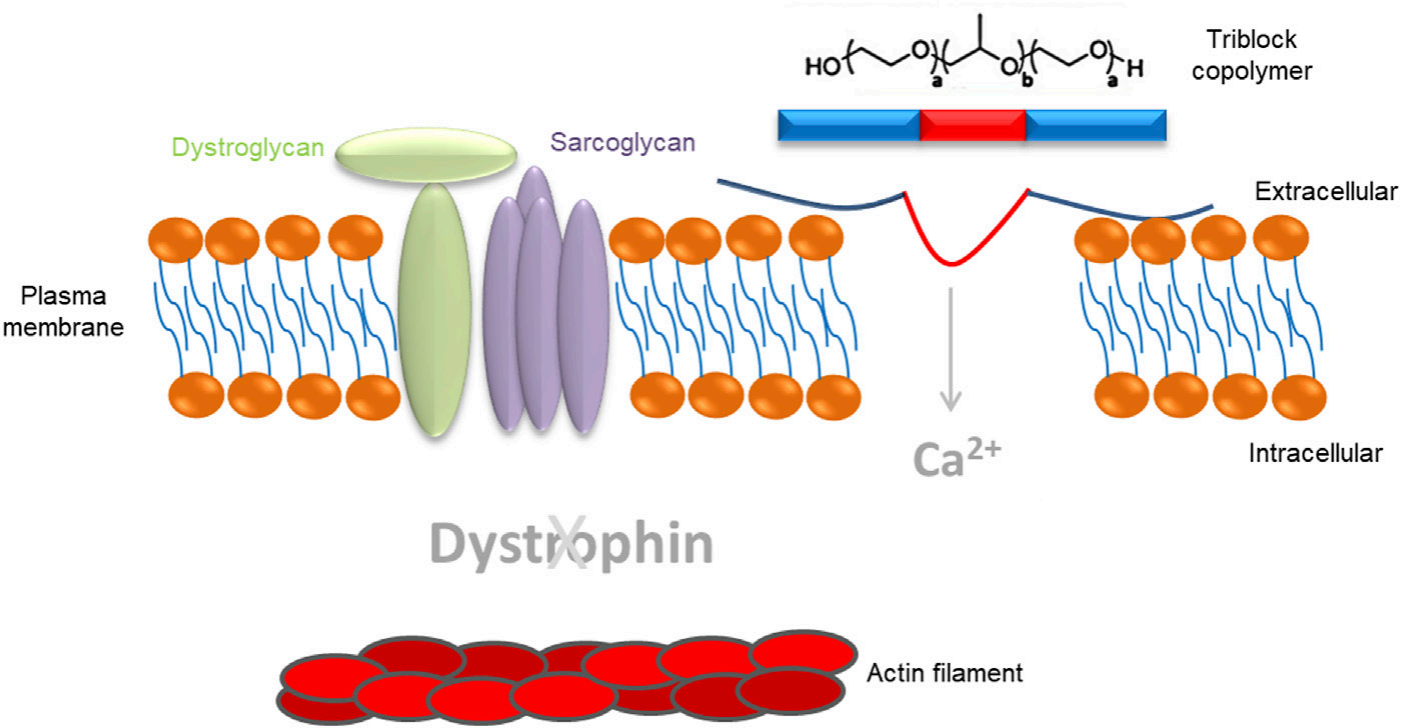
Copolymer-based muscle membrane stabilization of dystrophic muscle. Representation of copolymer-based stabilization of the damaged membrane via the interface of its hydrophobic PPO block (red) with stabilization by PEO blocks (blue) to prevent the entry of extracellular Ca^2+^ into the cell.

Poloxamers emerged from industrial applications in the 1940s. Recently, however, amphiphilic characteristics of poloxamer have shown a protective effect on the cell membrane. Poloxamer 188 (P188; PEO_75_-PPO_30_-PEO_75_, 8400 g/mol) is a non-ionic copolymer consisting of a triblock—a hydrophobic chain of poly(propylene oxide) (PPO) in the middle flanked by two hydrophilic poly(ethylene oxide) (PEO) chains on each side (Bates and Fredrickson, 1990). Studies using P188 have reported positive results in skeletal muscle fibers with electric injury (Lee et al., 1992; Lee et al., 1999; Collins et al., 2007), ischemia-reperfusion injuries (Hunter et al., 2010), heat injury (Merchant et al., 1998), radiation injury (Hannig et al., 1999), and sickle cell disease (Adams-Graves et al., 1997; Ballas et al., 2004). These results are due to the interactions between the phospholipid bilayer of the cells and the amphiphilic copolymer (Houang et al., 2017). Similar to these trials, DMD has been considered a potential candidate for the P188 treatment due to the fragile muscle cell membrane caused by dystrophin deficiency. In DMD, P188 is hypothesized to be beneficial in preventing cardiac myocyte damage induced by passive stretch and extracellular Ca^2+^ inflow triggered by micro-tears in the cell membrane (Yasuda et al., 2005) (Figure 3).

P188-treatment in isolated cardiac myocytes from mdx mice *in vitro* restored compliance and blocked susceptibility to stretch-induced Ca^2+^ overload (Yasuda et al., 2005). P188 delivery *in vivo* to mdx mice rapidly improved the geometry of the ventricles and suppressed the acute cardiac failure induced by a dobutamine stress test (Yasuda et al., 2005). P188 also prevented cardiomyopathy induced by isoproterenol in mdx mice (Spurney et al., 2011b). Similarly, systemic delivery of P188 for 8 weeks to dystrophic dogs (Golden retriever muscular dystrophy model) markedly reduced myocardial fibrosis, prevented left-ventricular remodeling, and inhibited the increase in cardiac Troponin I (TnI) and brain natriuretic peptide (BNP) in serum (Townsend et al., 2010). Furthermore, the direct application of P188 to isolated canine cardiac myocytes instantly restored poor myocyte compliance (Townsend et al., 2010).

For dystrophic skeletal muscle, the delivery method of P188 was found to be an important factor in the effectiveness of rescue of function. Interestingly, subcutaneous delivery of P188 showed protection against lengthening contraction-induced force loss, but this was not seen by intraperitoneal delivery. Furthermore, P188 significantly decreased baseline and lengthening contraction-induced membrane instability, as shown by reduced Evans Blue dye uptake in the mdx skeletal muscles (Houang et al., 2015). Skeletal muscles such as the diaphragm were also found to be protected by longer-term systemic delivery of P188 showing improved respiratory parameters, reduced centralized myonuclei, reduced variation in fiber size, and reduced collagen deposition (Markham et al., 2015).

Poloxamers can be manufactured in diverse forms by differing weight ratios of the PEO chain, molecular weight, and end group (Houang et al., 2018). Variations in hydrophilic and hydrophobic block lengths alter the hydrophobicity balance that determines the insertion of the molecule into the lipid bilayer of the cell and, thus, stabilization (Demina et al., 2005; Houang et al., 2017; Kim et al., 2017; Houang et al., 2018). For example, more hydrophobic poloxamers, such as P335 (PEO_38_–PPO_54_–PEO_38_), P333 (PEO_20_–PPO_54_–PEO_20_), and P181 (PEO_2_–PPO_30_–PEO_2_), insert into the liposomal membrane and eventually lyse the cell (Houang et al., 2018). Compared to P188, extended P188 (PEO_140_-PPO_44_-PEO_140_, 14,600 g/mol) also showed a robust protective effect at lower concentrations against the intracellular enzyme lactate dehydrogenase release induced by hypoosmotic stress (Houang et al., 2015). Furthermore, diblock copolymers (PEO_75_-PPO_15_) with a tert-butoxy end group (-C_4_) showed significant protection against lengthening contraction-induced force loss in mdx mice, whereas a hydroxyl end group (-H) did not show any protection (Houang et al., 2017). This finding showed that the hydrophobicity of the end group also directly influences interactions with the cell membrane and stabilization.

For membrane stabilizers in DMD, Phrixus Pharmaceuticals, Inc. has initiated a Phase 2 single-site, open-labeled trial for skeletal limb, cardiac, and respiratory muscle endpoints in non-ambulatory DMD patients (ClinicalTrials.gov Identifier: NCT03558958). Further research to determine the optimal structure-function of synthetic membrane-protecting copolymers will be useful in seeking a full therapeutic effect in DMD.

## 6 Non-dystrophin-based therapeutics

This section focuses on the genetic strategies related to the correction of or compensation for the lack of dystrophin via meaningful alternatives to direct dystrophin reconstitution. The advantage of dystrophin complementary approaches is that they may be applied to a number of MD patients, regardless of the type of mutation. Several pharmacological strategies to slow down the disease progress, such as those reducing inflammation or fibrosis, are also in development, and these have been reviewed by different groups (Spinazzola and Kunkel, 2016) and will not be covered in this review.

### 6.1 Utrophin modulation

Utrophin is a structural and functional autosomal paralog of dystrophin encoded by the UTRN gene (Love et al., 1989). This large cytoskeletal protein (395 kDa) shows sequence and structural similarity to dystrophin. Unlike dystrophin, utrophin is highly expressed in developing muscle and is localized at the sarcolemma in early human development (Love et al., 1989; Pons et al., 1993) when dystrophin is absent or present only at low levels. As the myofiber matures, utrophin levels decrease and become enriched at the neuromuscular junction (McNally, 2012).

Utrophin content is increased at the sarcolemma of skeletal muscle in DMD and BMD patients compared to healthy individuals (Weir et al., 2004; Arechavala et al., 2010; Mamchaoui, 2018). The baseline mild phenotype observed in the mdx mouse has been proposed to result from the efficient regeneration process in this model along with utrophin upregulation as a compensatory mechanism to mitigate the lack of dystrophin (Guiraud et al., 2015). This view is supported by the fact that mice deficient in both utrophin and dystrophin display a much more severe pathology compared to mdx mice (Deconinck et al., 1997). Furthermore, proof of principle that utrophin could play a role as a dystrophin surrogate comes from preclinical studies which established that an increase in utrophin protein levels in transgenic mdx mice prevented pathology, and this prevention was dependent on the amount of utrophin expressed (Tinsley et al., 1998). Studies in animal models have provided compelling evidence that utrophin functions in these scenarios to directly protect the muscle against dystrophin-deficient membrane instability. Because utrophin is an endogenous protein that can substitute for dystrophin, several approaches have been initiated to upregulate utrophin expression (Soblechero-Martín et al., 2021), including direct mechanisms, such as gene or protein replacement (Sonnemann et al., 2009), and indirect methods, such as transcriptional upregulation of the utrophin promoter or by stabilization of the protein or RNA (Michel et al., 2008).

Utrophin is expressed from at least two promoters, known as promoters A and B (Burton et al., 1999). Expression from promoter B is predominantly in endothelial cells in blood vessels, whereas expression from promoter A occurs in muscle and other tissues (Weir et al., 2002). Utrophin upregulation by stimulating promoter A activity is a promising pharmacological approach that has been extensively investigated. Thousands of small molecule candidates from drug libraries have been tested by high-throughput screening (HTS) assays to identify molecules acting at the utrophin A promotor site. Numerous small molecules, including heregulin (Krag et al., 2004), nabumetone (Moorwood et al., 2011), L-arginine (Voisin et al., 2005), and okadaic acid, have shown promising results at the preclinical level, with dose-dependent activation of the utrophin promoter. Ezutromid (SMTC1100) was the first orally bioavailable utrophin regulator that showed increased UTRN transcription (Soblechero-Martín et al., 2021). Pre-clinical results with the small molecule drug Ezutromid led Summit Therapeutics to initiate clinical trials as a potential treatment for DMD and BMD. Promising results from a Phase 1 healthy volunteer study showed that SMTC1100 is safe and well tolerated, with plasma levels achieved above those thought to be required to modulate utrophin (Ricotti et al., 2016). However, a Phase 2 clinical study (NCT02858362) failed to achieve both the primary (changes in leg muscle magnetic resonance parameters) and secondary (increased utrophin levels and decreased muscle damage) endpoints (Soblechero-Martín et al., 2021). Based on these results, Summit Therapeutics abandoned the development program of Ezutromid (Ricotti et al., 2016; Muntoni et al., 2019).

Direct utrophin protein replacement using recombinant full-length or truncated utrophin is another potential strategy to increase utrophin levels *in vivo*. The TAT protein transduction domain of the human immunodeficiency virus (HIV1) has been used to generate chimeric protein microutrophin (TAT-μUtr) and utrophin protein (TAT-Utr) (Sonnemann et al., 2009). Preclinical studies in mdx and dko mice were promising; however, further progress has not been reported. A similar pathway to micro-dys gene therapy has been pursued in the development of utrophin gene therapy. A number of preclinical studies using “micro-utrophin” (µUtrn) gene delivery have been reported recently, with some studies reporting restoration of the DGC, prevention of myofiber degeneration, normalization of serum CK levels, and improvement of muscle function (Ebihara et al., 2022). Furthermore, additional studies on double knockout (dko) mice and canine X-linked muscular dystrophy dogs have shown that µUtrn improves the severe pathological dystrophic phenotype in these models (Cerletti et al., 2003). One advantage to delivering utrophin is that it is less likely to elicit an immune response. It has been shown that viral gene delivery of dystrophin into DMD patients has been associated with an immune response, which limits the expression of dystrophin (Mendell et al., 2010). A protein such as utrophin, which is normally expressed in DMD patients, should not be associated with a similar immune response.

Utrophin, while highly similar to dystrophin, does not share all of the dystrophin binding domains. Therefore, it may not be able to fully substitute for dystrophin. Nonetheless, utrophin upregulation has been shown as a promising therapeutic approach, applicable to all DMD and BMD patients, irrespective of their dystrophin mutation. Many pathways involved in utrophin expression are currently being investigated. However, the amount of utrophin required by dystrophic patients to achieve a relevant clinical benefit remains to be determined. Further research is required before utrophin therapies can apply to DMD and BMD patients.

### 6.2 α7-Integrin upregulation

The α7β1 integrin protein is the predominant laminin-binding integrin in skeletal, cardiac, and vascular smooth muscle (Burkin and Kaufman, 1999). The integrin/laminin complex serves as a mechano-signaling anchor that binds laminin and links the ECM on the surface of muscle cells with the intracellular actin cytoskeleton (Hynes, 1992). α7 is present throughout the sarcolemma and is enriched at the myotendinous and neuromuscular junction. In particular, α7β1-integrin/laminin-211 plays a critical role in the functional integrity and maintenance of skeletal myoblasts and adult myofibers (Hodges et al., 1997; Mayer et al., 1997). α7 has been shown to be an important modifier of dystrophic symptoms, and defects in the components of this complex cause muscular dystrophy, illustrating the essential role of the α7 chain (Hodges et al., 1997; Guo et al., 2006). Interestingly, α7-integrin expression is increased at the sarcolemma in the mdx mouse and DMD patients (Hodges et al., 1997), demonstrating evidence that integrin upregulation may serve to functionally compensate for the lack of dystrophin. Moreover, knockout of both dystrophin and α7 integrin has been shown to produce a significantly more severe dystrophic phenotype, further supporting the evidence of a compensatory role for α7 integrin for dystrophin (Rooney et al., 2006).

Burkin et al. (2001, 2005) showed that transgenic overexpression of α7-integrin in dystrophin/utrophin double knockout mice (*mdx*/utrn^−/−^) alleviates pathology, extends viability and mobility, and reduces kyphosis. In addition, increased expression of α7 in mdx mice significantly protected against loss of force, reversed muscle pathology, and stabilized sarcolemmal integrity (Heller et al., 2013). Importantly, eightfold overexpression of α7-integrin does not demonstrate detectable toxicity or disruption to global gene expression profiles (Liu et al., 2008). Therefore, similar to utrophin, small compound screens for α7-integrin modulators appear to be a viable approach (Gurpur et al., 2009).

Laminin-111 is another potential candidate of interest (Rooney et al., 2009a). Injection of laminin-111 protein in the mdx mouse increased the expression of α7-integrin, stabilized the sarcolemma, and protected muscle from exercise-induced damage (Rooney et al., 2009a; 2009b). Conversely, transgenic expression of the laminin α1 chain to enhance heterotrimer formation of laminin-111 in the mdx mouse reported no improvement of the dystrophic symptoms (Gawlik et al., 2011), showing that further studies are necessary to validate the functionality of laminin-111 protein therapy in DMD.

### 6.3 Myostatin, follistatin, and other muscle growth strategies

Myostatin is a transforming growth factor-β-superfamily member that acts as a negative regulator of skeletal muscle growth (Mcpherron et al., 1997). The biological relevance of myostatin was addressed in mice through gene overexpression studies using normal or dominant-negative forms of the protein, systemic administration of myostatin protein or inactivating antibody, and gene inactivation (Zhu et al., 2000; Yang et al., 2001; Nishi et al., 2002; Grobet et al., 2003; Walsh and Celeste, 2005; Reisz-porszasz et al., 2022). Regardless of the approach used to induce myostatin inhibition, studies have shown functional improvement of the dystrophic muscle, such as increasing body weight, muscle mass, and muscle size, along with a significant decrease in muscle degeneration. Based on these studies and others carried out in cultured cells, the general consensus is that myostatin regulates postnatal muscle-fiber size by maintaining satellite cells in a quiescent state and inhibiting protein synthesis. Myostatin also regulates the number of muscle fibers during development by blocking the proliferation and differentiation of myoblasts (Lee, 2004; Tobin and Celeste, 2005).

However, some concerns have been raised about whether myostatin inhibition leads to a truly healthy muscle, as an exercise in myostatin-deficient cattle leads to early exhaustion. Moreover, it can even be deleterious, as disused muscle atrophy is markedly more severe in the context of myostatin deficiency (Mcmahon et al., 2022). Nonetheless, considering the effect of myostatin on muscle growth, inhibition of myostatin has been considered a therapeutic target in the treatment of muscle-degenerative and wasting conditions, such as muscular dystrophies, and clinical trials have been designed to increase muscle mass and strength in several of the most common forms of adult muscular dystrophy. Nevertheless, clinical trials in humans have been disappointing owing to a lack of improvement in muscle strength (Wagner et al., 2008) or adverse effects (Attie et al., 2013). Despite these negative results, other clinical trials based on blocking myostatin activity have been initiated (Mulivor et al., 2014; Pfizer, 2014).

## 7 Clinical trials and approved therapies

A growing number of pharmaceutical companies and startups have directed efforts toward DMD therapeutics. Many challenges remain, and the problem of delivery to all muscles of the body needs to be resolved. Nevertheless, there is little doubt that although a cure remains elusive, there has been a rapid expansion in the number of treatments entering clinical trials that have the potential to provide a significant clinical impact on the quality of life of DMD patients (ClinicalTrials.gov). Some of these approaches have received regulatory approval in the USA, Europe, and Japan (Mercuri et al., 2019). The increase in the number of patient registries and the ability to link natural histories significantly enhance opportunities to examine inter-individual differences to best evaluate disease progression/regression. These opportunities will greatly facilitate the emerging field of personalized/combinatory therapies, which represent the future of effective DMD treatments and will set the stage for other rare disease therapeutics.

Gene therapy has been elevated in the DMD field due to the promise that this approach could “stably” correct DMD defects via restoring dystrophin throughout the body (Gregorevic et al., 2006; Chamberlain and Chamberlain, 2017). Adeno-associated virus- (AAV) mediated gene therapies have advanced significantly since their clinical trial debut in 2003 (Kuzmin et al., 2021). Several studies have demonstrated the body-wide expression and therapeutic effect of this approach following intravascular AAV microdystrophin delivery (Mendell et al., 2021). Based on the many positive efficacy reports from experiments in the mdx mouse model and golden retrievers with muscular dystrophy (GRMD dog model) (Birch et al., 2023), four microdystrophin constructs have moved forward and are currently being investigated in clinical trials (Figure 1) (Table 2). The clinical trials were initiated in the United States in December 2017 and are now ongoing in Europe.

Clinical trial PF-06939926 is an AAV9-mediated transfer of microdystrophin currently tested in a Pfizer Phase 1b open-label clinical trial evaluating dose, safety, and tolerability of a single IV infusion of microdystrophin in ambulatory and non-ambulatory DMD patients (NCT03362502 and NCT04281485) (Table 2). PF-06939926 has been tested in men with DMD, ages 4 and older, at 21 sites, and two doses have been used: a low dose of 1 × 10^14^ vector genomes (vg)/kg) and a higher dose of 2 × 10^14^ vg/kg. Early data from five of six boys showed some evidence of improvement (or at least no decline) in the NorthStar Ambulatory Assessment (NSAA), compared with participants given a placebo during previous clinical trials. Side effects and serious adverse events were reported in some patients. Tragically, a death of a patient was reported, which led to the trial being put on hold due to questions about Pfizer’s potency tests. Recently, the FDA announced the removal of the clinical hold after the company had addressed the agency’s request regarding a potency assay and implementation of a protocol amendment.

SGT-001, an AAV9-mediated transfer of microdystrophin from Solid Biosciences, is also being evaluated in a phase I clinical trial, IGNITE DMD (NCT03368742) (Table 2). After a suspension due to a serious adverse effect in one patient, the study was recently re-activated with an amended clinical protocol and using SGT-001 manufactured with a second-generation process (Solid Bioscience). A clinical trial by Sarepta Therapeutics, Inc. is ongoing to investigate the safety and efficacy of the IV infusion of rAAVrh74.MHCK7 microdystrophin (SRP-9001), in a first open-label phase I/II trial (NCT03375164). Some evidence of 12-week dystrophin expression and a good safety profile was communicated from the first 11 participants enrolled in Study SRP-9001–103 (NCT04626674), another open-label Phase I study being conducted in partnership with Roche (Sarepta therapeutics).

Overall, to date, clinical trials have accrued preliminary results that have unfortunately demonstrated a lack of clinically meaningful success and missed milestones for efficacy (Mullard, 2021a). Thus far, the human trial data do not report the same success reported in animal works, indicating this promising approach requires significant further enhancement (Mullard, 2021b).

There is a clear need for caution as several serious treatment-related adverse events have been reported. Hepatic transaminitis, classed as severe, was reported in participants in trials in DMD, following SGT001 (Wagner et al., 2021) and rAAVrh74.MHCK7.microdystrophin (NCT03375164) (Mendell et al., 2020). One incidence of vomiting requiring hospital admission was reported as a severe adverse event with the PF-06939926 microdystrophin product (NCT03362502) (Belluscio et al., 2021). Moreover, two patients receiving rAAVrh74.MHCK7.microdystrophin (NCT03375164) experienced severe rhabdomyolysis (Today MDN, 2019; Novack et al., 2021). Significant issues remain to be considered, such as pre-existing DMD population immunity to certain AAV serotypes, the efficacy of the specific truncated micro- or mini-dystrophin variants used, and re-administration challenges and serotype switching considerations.

Consequently, other approaches aim to produce nearly full-length dystrophin by targeting only the region that encompasses the mutation in an effort to bypass the genetic defect. During the last decade, numerous approaches have been developed to directly correct genetic defects at the RNA level (Hanson et al., 2021), as discussed previously. In the case of dystrophin, such approaches are designed to either restore the expression of a full-length transcript or a nearly full-length *DMD.*

Ataluren (Translarna™, previously known as PTC124) is an orally bioavailable small molecule developed by PTC Therapeutics designed to enable the formation of a functioning protein in patients with genetic disorders caused by a nonsense mutation (Politano, 2021). Ataluren has demonstrated its efficacy *in vivo* using the mdx mouse model (Welch et al., 2007). However, laboratories failed to replicate this finding, questioning the specificity of Ataluren against stop codons (Auld et al., 2009; McElroy et al., 2013). Nonetheless, supported by evidence for functional improvement in a cell model of Hurler syndrome (Goldmann et al., 2012) and in mouse models of nonsense mutation-associated cystic fibrosis (Kerem et al., 2014), Ataluren advanced to clinical trials and was granted conditional marketing authorization by EMA in 2014 (https://www.ema.europa.eu/en/medicines/human/EPAR/translarna) for DMD ambulatory patients and those aged ≥2 years (Table 3). To date, two randomized, double-blind, placebo-controlled trials of Ataluren in DMD patients have been conducted: a Phase IIb trial (NCT00592553) on 174 randomized patients and a Phase III trial (NCT01826487) on 230 randomized patients (Table 3). In both trials, Ataluren was well-tolerated (Campbell et al., 2020). However, the trials failed to achieve the 48-week primary endpoint of improved distance walked in the 6-min walk test (SMWT) compared with patients who received placebo treatment. Nonetheless, these trials reported a trend of therapeutic efficacy, specifically a 29-m increase in the SMWT and an improvement in timed function tests in those who received Ataluren compared with placebo (Campbell et al., 2020; Politano, 2021). Unfortunately, very little evidence exists to inform the question of whether Ataluren displays any degree of efficiency or delivers a benefit in the heart.

**TABLE 3:** Clinical trials using stop codon read-through and exon skipping for DMD therapy in 2023.

Drug name	Description	Company	Delivery route	Current stage	Clinical trial number
Stop codon read-through					
Ataluren	Nonsense suppression	PTC Therapeutics	Oral	Phase III	NCT01557400
Exon skipping					
Eteplirsen (AVI-4568)	PMO morpholino targeting exon 51	Sarepta Therapeutics	Intravenous	Phase II	NCT03218995
				Phase II	NCT04179409
				Phase III	NCT03992430
				Phase III	NCT03985878
Golodirsen (SRP4053)	PMO morpholino targeting exon 53	Sarepta Therapeutics	Intravenous	Phase II	NCT04179409
				Phase III	NCT02500381
				Phase III	NCT03532542
Casimersen (SRP4053)	PMO morpholino targeting exon 45	Sarepta Therapeutics	Intravenous	Phase II	NCT04179409
Viltolarsen (NCNP-01)	PMO morpholino targeting exon 53	Nippon Shinyaku Co. Ltd	Intravenous	Phase II	NCT03167255
Drisapersen (PRO051)	2′-O-methyl PS Targeting exon 51	BioMarin Pharmaceutical Inc	Intravenous	Extension	NCT02636686

A number of chemical versions of AONs exist to be applied for exon-skipping, including 2^′^-*O*-methyl-modified RNA, phosphorodiamidate morpholino oligomers (PMO), and tricycloDNA antisense. All of them have been used *in vitro* and *in vivo* in mouse and dog models and demonstrated efficient skipping that resulted in histology and functional improvements (Niks and Aartsma-Rus, 2017), as previously discussed. Several PMO compounds are in development to target the exons that represent the highest proportions of deletions amenable to exon-skipping. Eteplirsen (marketed under the trade name Exondys 51) is a morpholino ASO from Sarepta Therapeutics designed to mask a splice acceptor sequence in exon 51 of the dystrophin gene, thereby promoting exon skipping and restoration of the reading frame in the 13% of patients with amenable frame-shifting mutations. This was the first drug approved by the FDA as a specific DMD therapy. Patients’ muscle biopsies after approximately a year of systemic dosing showed modest efficacy in the production of dystrophin protein expression (∼1%), which is considered to be inadequate to confer significant clinical benefits (NCT01396239, NCT01540409, NCT02255552) (Table 3) (Mendell et al., 2016; Niks and Aartsma-Rus, 2017).

Regardless, the drug was approved, and efforts are underway to develop more effective oligonucleotide therapies. Tissue penetration and longevity of PMOs have remained an ongoing concern. To address these shortcomings, other delivery systems for AONs are currently being investigated to identify the modifications that can optimize cellular penetration and systemic safety, with a major focus on PPMOs (Nguyen and Yokota, 2019). Additionally, multiple other exon-skipping AONs are being actively developed, with the FDA recently giving approval for Golodirsen (SRP-4053) and Viltolarsen, designed to skip exon 53, and Casimersen (SRP-4045), designed to skip exon 45 (Table 3). So far, data collected from DMD patients show that PMO AONs are well tolerated and safe in DMD following weekly intravenous (IV) administration (Frank et al., 2020; Komaki et al., 2020; Wagner et al., 2021).

## 8 Conclusion

The identification of the dystrophin gene as the cause of DMD has led to improved diagnosis while providing deep insights into the biochemistry and cellular physiology of the striated muscle cytoskeleton-membrane-ECM interface. The preceding decades have been marked by careful mechanistic studies aiming to tease apart the molecular processes disrupted by dystrophin loss in an effort to understand and correct the underlying pathology of DMD. These insights have greatly impacted DMD diagnosis and care, including the opportunity to develop novel, personalized, and effective therapeutic strategies to prolong and improve the quality of life of patients with DMD.

## 9 Future directions

The future of heart/skeletal muscle therapy in DMD will likely require a combination of approaches to achieve optimal outcomes, including a therapeutic approach to correct the genetic defect and target the secondary effects caused by the lack of dystrophin. Advances in technology have made many of the problems with therapeutic approaches more tractable. Despite that, two crucial and challenging issues shared by all new drugs are delivery and targeting. Finding an appropriate vehicle is critical to consistently reach the proper drug target at minimal, effective dosing. This is especially significant considering the enormous healthcare costs associated with these proposed therapies. Nonetheless, there is little doubt that although a cure remains elusive, there has been a rapid expansion in the number of treatments entering clinical trials that have the potential to provide a significant clinical impact on the quality of life for DMD patients. This turning point in the development of DMD therapies marks the beginning of a new mission for correcting monogenic muscular disorders. In turn, these approaches set an example for research progress for other related disorders.
